# Quantitative ethnopharmacological documentation and molecular confirmation of medicinal plants used by the *Manobo* tribe of Agusan del Sur, Philippines

**DOI:** 10.1186/s13002-020-00363-7

**Published:** 2020-03-05

**Authors:** Mark Lloyd G. Dapar, Grecebio Jonathan D. Alejandro, Ulrich Meve, Sigrid Liede-Schumann

**Affiliations:** 1 The Graduate School and Research Center for the Natural and Applied Sciences, University of Santo Tomas, España Boulevard, 1015 Manila, Philippines; 2 College of Science, University of Santo Tomas, España Boulevard, 1015 Manila, Philippines; 3 Department of Plant Systematics, University of Bayreuth, Universitätsstr. 30, 95440 Bayreuth, Germany

**Keywords:** *Agusan Manobo*, Cultural importance value, Ethnopharmacology, Mindanao, Molecular confirmation, Use diversity

## Abstract

**Background:**

The Philippines is renowned as one of the species-rich countries and culturally megadiverse in ethnicity around the globe. However, ethnopharmacological studies in the Philippines are still limited especially in the most numerous ethnic tribal populations in the southern part of the archipelago. This present study aims to document the traditional practices, medicinal plant use, and knowledge; to determine the relative importance, consensus, and the extent of all medicinal plants used; and to integrate molecular confirmation of uncertain species used by the *Agusan Manobo* in Mindanao, Philippines.

**Methods:**

Quantitative ethnopharmacological data were obtained using semi-structured interviews, group discussions, field observations, and guided field walks with a total of 335 key informants comprising of tribal chieftains, traditional healers, community elders, and *Manobo* members of the community with their medicinal plant knowledge. The use-report (UR), use categories (UC), use value (UV), cultural importance value (CIV), and use diversity (UD) were quantified and correlated. Other indices using fidelity level (FL), informant consensus factors (ICF), and Jaccard’s similarity index (JI) were also calculated. The key informants’ medicinal plant use knowledge and practices were statistically analyzed using descriptive and inferential statistics.

**Results:**

This study enumerated the ethnopharmacological use of 122 medicinal plant species, distributed among 108 genera and belonging to 51 families classified in 16 use categories. Integrative molecular approach confirmed 24 species with confusing species identity using multiple universal markers (ITS, *mat*K, *psb*A-*trn*H, and *trn*L-F). There was strong agreement among the key informants regarding ethnopharmacological uses of plants, with ICF values ranging from 0.97 to 0.99, with the highest number of species (88) being used for the treatment of abnormal signs and symptoms (ASS). Seven species were reported with maximum fidelity level (100%) in seven use categories. The correlations of the five variables (UR, UC, UV, CIV, and UD) were significant (*r*_s_ ≥ 0.69, *p* < 0.001), some being stronger than others. The degree of similarity of the three studied localities had JI ranged from 0.38 to 0.42, indicating species likeness among the tribal communities. Statistically, the medicinal plant knowledge among respondents was significantly different (*p* < 0.001) when grouped according to education, gender, social position, occupation, civil status, and age but not (*p* = 0.379) when grouped according to location. This study recorded the first quantitative ethnopharmacological documentation coupled with molecular confirmation of medicinal plants in Mindanao, Philippines, of which one medicinal plant species has never been studied pharmacologically to date.

**Conclusion:**

Documenting such traditional knowledge of medicinal plants and practices is highly essential for future management and conservation strategies of these plant genetic resources. This ethnopharmacological study will serve as a future reference not only for more systematic ethnopharmacological documentation but also for further pharmacological studies and drug discovery to improve public healthcare worldwide.

## Introduction

The application of traditional medicine has gained renewed attention for the use of traditional, complementary, and alternative medicine (TCAM) in the developing and industrialized countries [1, 2]. Conventional drugs these days may serve as effective medicines and therapeutics, but some rural communities still prefer natural remedies to treat selected health-related problems and conditions. Medicinal plants have long been used since the prehistoric period [3], but the exact time when the use of plant-based drugs has begun is still uncertain [4]. The WHO has accounted about 60% of the world’s population relying on traditional medicine and 80% of the population in developing countries depend almost entirely on traditional medical practices, in particular, herbal remedies, for their primary health care [5]. Estimates for the numbers of plant species used medicinally worldwide include 35,000–70,000 [6] with 7000 in South Asia [7] comprising ca. 6500 in Southeast Asia [8, 9]. In the Philippines, more than 1500 medicinal plants used by traditional healers have been documented [10], and 120 plants have been scientifically validated for safety and efficacy [11]. Of all documented Philippine medicinal plants, the top list of medicinal plants used for TCAM has been enumerated by [12]. Most of these Philippine medicinal plants have been evaluated to scientifically validate folkloric claims like the recent studies of [13–20].

Because of the increasing demand for drug discovery and development of medicinal plants, the application of a quantitative approach in ethnobotany [21] and ethnopharmacology [22] has been rising continuously in the last few decades including multivariate analysis [23]. However, few studies of quantitative ethnobotanical research were conducted despite the rich plant biodiversity and cultural diversity in the Philippines. In particular, the *Ivatan* community in Batan Island of Luzon [24] and the *Ati Negrito* community in Guimaras Island of Visayas [21] have been documented, while Mindanao has remained less studied. Despite the richness of indigenous knowledge in the Philippines, few ethnobotanical studies have been conducted and published [25].

The Philippines is culturally megadiverse in diversity and ethnicity among indigenous peoples (IPs) embracing more than a hundred divergent ethnolinguistic groups [26, 27] with known specific identity, language, socio-political systems, and practices [28]. Of these IPs, 61% are mainly inhabiting Mindanao, followed by Luzon with 33%, and some groups in Visayas (6%) [29]. One of these local people and minorities is the indigenous group of *Manobo*, inhabiting several areas only in Mindanao. They are acknowledged to be the largest Philippine ethnic group occupying a wide area of distribution than other indigenous communities like the Bagobo, Higaonon, and Atta [30]. The *Manobo* (“river people”) was the term named after the “Mansuba” which means river people [19], coined from the “man” (people) and the “suba” (river) [31]. Among the provinces dwelled by the *Manobo*, the province of Agusan del Sur is mostly inhabited by this ethnic group known as the *Agusan Manobo*. The origin of *Agusan Manobo* is still uncertain and immemorial; however, they are known to have Butuano, Malay, Indonesian, and Chinese origin occupying mountain ranges and hinterlands in the province of Agusan del Sur [32].

*Manobo* indigenous peoples are clustered accordingly, occupying areas with varying dialects and some aspects of culture due to geographical separation. Their historic lifestyle and everyday livelihood are rural agriculture and primarily depend on their rice harvest, root crops, and vegetables for consumption [33]. Some *Agusan Manobo* are widely dispersed in highland communities above mountain drainage systems, indicating a suitable area for their indigenous medicinal plants in the province [34]. Every city or municipality is governed with a tribal chieftain known as the “Datu” (male) or “Bae” (female) with his or her respective tribal healer “Babaylan” and the tribal leaders “Datu” of each barangay (village) leading their community. Their tribe has passed several challenges over the years but has still maintained to conserve and protect their ancestral domain to continually sustain their cultural traditions, practices, and values up to this present generation. This culture implies that there is rich medicinal plant knowledge in the traditional practices of *Agusan Manobo*, but their indigenous knowledge has not been systematically documented. Furthermore, there are no comprehensive ethnobotanical studies of medicinal plants used among the *Manobo* tribe in the Philippines to date.

Documenting the ethnomedicinal plant use and knowledge, and molecular confirmation of species using integrative molecular approach will help in understanding the true identity of medicinal plants in the treatment of health-related problems of the people of Agusan del Sur. This will also help the entire *Agusan Manobo* community to implement conservation priorities of their indigenous plant species. Furthermore, the provincial government of Agusan del Sur may enforce the proper utilization of their plant resources from IPs. Ideas and knowledge about ethnomedicinal use and practices of medicinal plants give credence to the traditional methods and preparation of herbal medicine by ethnic groups.

Despite the limited funds and qualified personnel in the region, it is very relevant to recognize the role of ethnopharmacology and species identification in the conservation of these plant genetic resources with medicinal properties. With the introduction of the application of molecular barcodes for species identification by [35], the problem of unauthenticated medicinal species can now be resolved [19, 36–43].

Significantly, researchers have recently developed the application of ethnopharmacological study into a quantitative approach with measuring values and indices to quantify the relationship between plant species and humans [44–48].

This study, therefore, aims to (1) conduct quantitative ethnopharmacological documentation of traditional therapy, (2) evaluate the medicinal plant use and knowledge, and (3) utilize integrative molecular approach for species confirmation of medicinal plants used by the *Manobo* tribe in Agusan del Sur, Philippines.

## Materials and methods

### Study area

**Fig. 1:**
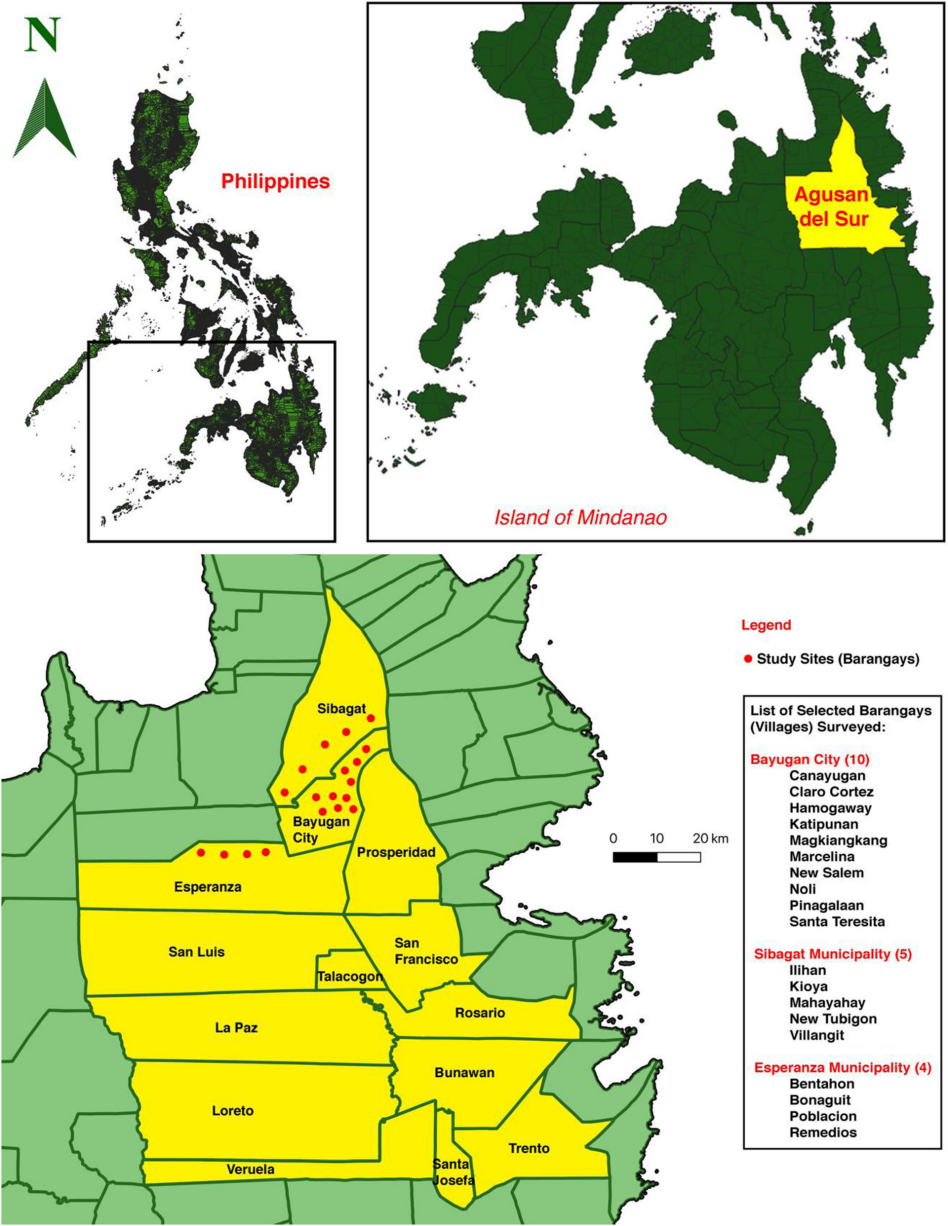
Study sites (barangays) from the only city (Bayugan), and the two selected municipalities (Esperanza and Sibagat) in the province of Agusan del Sur

Fieldwork was conducted in the province of Agusan del Sur, Philippines (8° 30′ N 125° 50′ E), bordered from the north by Agusan del Norte, to the south by Davao del Norte, and from the west by Misamis Oriental and Bukidnon, to the east by Surigao del Sur. Agusan del Sur is bounded with mountain ranges from the eastern and western sides forming an elongated basin or valley in the center longitudinal section of the land. The province is subdivided into 13 municipalities (from the largest to smallest land area): La Paz, Esperanza, Loreto, San Luis, Talacogon, Sibagat, Prosperidad, Bunawan, Trento, Veruela, Rosario, San Francisco, and Sta. Josefa; and the only component city, the City of Bayugan (Fig. 1). Forestland comprises almost two thirds (74%) of the province of Agusan del Sur, while alienable and disposable (A&D) areas constitute around one-third (26%) of the total land area [49]. Every city or municipality has a respective community hospital and health center with limited doctors and rural health workers. Typically, local people only visit the hospitals or health centers for surgical and obstetric emergencies. Most residents rely on their medicinal plants for disease treatment and medication due to cost and poor access to healthcare services. This study purposively covered areas of selected city and municipalities (Bayugan, Esperanza, and Sibagat) for accessibility, availability, and security reasons to barangays (villages) with Certification of Ancestral Domain Title (CADT) as endorsed by the National Commission on Indigenous Peoples—CARAGA Administrative Region (NCIP-CARAGA).

### Sampling and interview

Fieldwork was undertaken from March 2018 to May 2019. It consisted of obtaining free prior informed consents, observing rituals, acquiring resolutions, certifications, and permits, conducting semi-structured interviews, focus group discussions, plant and field observations, and medicinal plant collections in selected barangays (villages) of Bayugan, Sibagat, and Esperanza (Fig. 1). This study was initiated in coordination with the local government unit (LGU), NCIP-LGU, and Provincial Environment and Natural Resources Office (PENRO) of Agusan del Sur. Consultation meetings and discussions were carried out together with the concerned parties (tribal leaders, tribal healers, and NCIP officers) to discuss research intent as purely academic and to acquire mutual agreement and respect to conduct this study. As approved, the research intent was certified through resolution and certification duly signed by the tribal council of elders following the by-laws of NCIP for the welfare and protection of indigenous peoples, and finally certified by NCIP-CARAGA.

Ethnopharmacological data were collected through semi-structured interviews with *Manobo* key informants through purposive and snowball sampling who were certified *Agusan Manobo*. A sampling of these key informants was coordinated with the provincial and local government administration together with the assistance of the tribal leaders and NCIP focal persons in every city or municipality to each of the barangays in selecting those who have knowledge of their medicinal plants and practices. The respective barangay tribal leaders assisted interviews among respondents with no appointments made prior to the visits. The semi-structured questionnaire used was modified and adapted from the Traditional Knowledge Digital Library (TKDL) template, as suggested by the Department of Health—Philippine Institute of Traditional and Alternative Health Care (DOH-PITAHC) (see Additional file 1). The Ethics Review Committee of the Graduate School, University of Santo Tomas (USTGS-ERC), approved the study and the questionnaire used with a valid translation to Manobo dialect (*Minanubu*) with the help of a community member and NCIP officer. It has series of questions about the common health problems encountered by the respondents; the actions undertaken to address such problems; the medicinal plants they used (local or vernacular name); the plant’s part(s) used, forms, modes, quantity or dosage, and frequency of administration; the source or transfer of knowledge; and the experienced adverse or side effects. Interviews were accompanied by nurses and allied workers as coordinated by the rural health center to verify reported diseases accurately by the informants.

Meetings and focus group discussions were also performed to review the accuracy of acquired data among the respondents with the help of guided questions among the tribal council of elders comprising the NCIP-recognized indigenous peoples mandatory representatives (IPMRs), the tribal chieftains, the tribal healers, and the respective tribal leaders of every barangay tribal communities together with the NCIP officer.

### Plant collection and identification

The collection of plant specimens was conducted through guided field walks with the aid of the traditional healers, expert plant gatherers, and members within the tribal community. The plant habit, habitat, morphological characteristics, vernacular names, and some indigenous terms of their uses were documented. Leaf samples were placed in zip-locked bags containing silica gel for molecular analysis [50] in preparation for further molecular confirmation. Voucher specimens were deposited in the University of Santo Tomas Herbarium (USTH). Putative plant identification using vernacular names was compared to the reference of local names, *Dictionary of Philippines Plant Names* by [51]. Plant identification was assisted by Mr. Danilo Tandang, a botanist and researcher at the National Museum of the Philippines. Specimens unidentifiable by morphology were selected for molecular confirmation. All scientific names were verified and checked for spelling and synonyms and family classification using *The Plant List* [52], *World Flora Online* [53], The *International Plant Names Index* [54], and *Tropicos* [55]. The occurrence, distribution, and species identification were further verified using the updated *Co’s Digital Flora of the Philippines* [56].

### DNA extraction, amplification, and sequencing

Collected plant specimens with insufficient material for identification due to lack of reproductive parts and unfamiliarity were subjected to molecular confirmation. The total genomic DNA was extracted from the silica gel-dried leaf tissues of samples following the protocols of DNeasy Plant Minikit (Qiagen, Germany). The ITS (nrDNA), *mat*K, *trn*H-*psb*A, and *trn*L-F (cpDNA) markers were used for this study. Primer information and PCR conditions used for amplification using Biometra T-personal cycler (Germany) can be found in Table 1 for future parameter reference. PCR amplicons were checked on a 1% TBE agarose to inspect for the presence and integrity of DNA. Amplified products were sent to Eurofins Genomics (Germany) for DNA sequencing reactions. Sequences were then assembled and edited using Codon Code Aligner v4.1.1. All sequences were then evaluated and compared using BLAST*n* search query available in the GenBank (www.ncbi.nlm.nih.gov). The BLAST*n* method estimates the reliability of species identification as a sequence similarity search program to determine the sequence of interest [62] regardless of the age, plant part, or environmental factors of the sample [63].

**Table 1:** Gene regions, primers and amplification protocols used for polymerase chain reaction

Gene region	Primer name	Reference	Primer sequence (5′ ➔ 3′)	PCR Protocol
ITS (ITS1, 5.8S gene, and ITS2)	*p17*F	[57]	5′-*CTACCGATTGAATGGTCCGGTGAA*-3′	94 °C 5 min; 28 cycles of 94 °C 1 min, 48 °C 1 min, 72 °C 1 min; 72 °C 7 min; 10 °C paused
	*26*S*-82*R		5′-*TCCCGGTTCGCTCGCCGTTACTA*-3′	
	*5*	[58]	5′-*GGAAGTAAAAGTCGTAACAAGG*-3′	94 °C 5 min; 30 cycles of 94 °C 1 min, 55 °C 1 min, 72 °C 1 min, 45 s; 72 °C 10 min; 10 °C paused
	*4*		5′-*TCCTCCGCTTATTGATATGC*-3′	
*mat*K	*3*F*_kim*F	[59]	5′-*CGTACAGTACTTTTGTGTTTACGAG*-3′	98 °C 45 s; 35 cycles of 98 °C 10 s, 52 °C 30 s, 72 °C 40 s; 72 °C 10 min; 10 °C paused
	*I*R*_kim*R		5′-*ACCCAGTCCATCTGGAAATCTTGGTTC*-3′	
*psb*A-*trn*H	*psb*A*_*F	[60]	5′-*GTTATGCATGAACGTAATGCTC*-3′	95 °C 4 min; 35 cycles of 94 °C 30 s, 55 °C 1 min, 72 °C 1 min; 72 °C 10 min; 10 °C paused
	*trn*H*_*R		5′-*CGCGCATGGTGGATTCACAATCC*-3′	
*trn*L-F	*c*	[61]	5′-*CGAAATCGGTAGACGCTACG*-3′	94 °C 3 min; 30 cycles of 93 °C 1 min; 55 °C 1 min, 72 °C 2 min; 10 °C paused
	*f*		5′-*ATTTGAACTGGTGACACGAG*-3′	

### Quantitative ethnopharmacological analysis

The use-report (UR) is counted as the number of times a medicinal plant is being used in a particular purpose in each of the categories [21, 24]. Only one use-report was counted for every time a plant was cited as being used in a specific disease or purpose and even multiple disease or purpose under the same category [64]. Multiple use-reports were counted when at least two interviewees cited the same plant for the same disease or purpose. The use value (UV) developed by [45] is used to indicate species that are considered highly important by the given population using the following formula: UV = (ΣUi)/*N*, where Ui is the number of UR or citations per species and *N* is the total number of informants [47, 48]. High UV implies high plant use-reports relative to its importance to the community and vice versa. However, it does not determine whether the use of the plant is for single or multiple purposes [21, 24]. The relative importance of the plants was also determined by calculating the cultural importance value (CIV) by using the formula: CIV = Σ[(ΣUR)/*N*], where UR is the number of use-reports in use category and *N* is the number of informants reporting the plant [48]. The use diversity (UD) of each medicinal plant used was determined using the Shannon index of uses as calculated with the R package *vegan* [65].

The ICF introduced by [66] was used to analyze the degree of informants’ agreement based on their medicinal plant knowledge in each of the categories [21, 24]. This is computed using the formula: ICF = (Nur − Nt)/(Nur − 1), where Nur is the number of UR in each category, and Nt is the number of species used for a particular category by all informants. Fidelity level (FL) developed by [67] is calculated using the formula: FL (%) = (Ip/Iu) × 100, where Ip is the number of informants who independently suggested a given species for a particular disease, and Iu is the total number of informants who mentioned the plant for any use or purpose regardless of category. The maximum value (1.00) means a high degree of informant agreement showing the effectiveness of medicinal plants in each ailment category [68]. However, a minimum value (0.00) implies no information exchange among the informants [69]. Jaccard’s similarity index (JI) by [70] was calculated to evaluate the similarity of medicinal plant species among the three studied areas. The formula of JI is represented as follows: *J* = *C*/(*A* + *B*), where *A* is the number of species found in habitat a, *B* is the number of species found in habitat b, and *C* is the number of common species found in habitats a and b. The number species present in either of the habitats is given by *A* + *B* (Jaccard).

### Statistical tools

The plant URs were computed and analyzed using IBM SPSS Statistics software v.23 [71]. Descriptive and non-parametric inferential statistics Mann-Whitney *U* and Kruskal-Wallis tests were employed to test for significant differences at 0.01 level of significance. These two statistical analyses measure and compare the medicinal plant use and knowledge of informants when grouped according to location, education, gender, social position, occupation, civil status, and age. The basic values and indices (UR, UC, UV, CIV, UD) were correlated using the Spearman correlation coefficient to compare variables that are not distributed normally.

### Integrative molecular confirmation

Selected plant samples unidentifiable by morphology were subjected to an integrative molecular identification approach as previously recommended by [42] for accurate species identification of plant samples. Selected plant samples were compared with the available morphological characteristics, interview data on vernacular names and traditional knowledge, determining scientific names based on reference of local names using the *Dictionary of Philippines Plant Names* by [51], and utilizing multiple molecular markers, ITS (nrDNA), *mat*K, *trn*H-*psb*A, and *trn*L-F (cpDNA) for sequencing and BLAST matching. Two sequence similarity-based methods using BLAST [72] were applied for molecular confirmation. BLAST similarity-based identification was adapted from the study of [42] with a slight modification. This identification involved using the simple method taking the top hits and optimized approach. All successfully sequenced samples were sequentially queried using megablast [72] online at NCBI nucleotide BLAST against the nucleotide database. For the simple method, all top hits within a 5-point deviation down of the max score were considered. If the max score (− 5 points) showed only a single species, then a species level identification was assigned. On the other hand, if the max score (− 5 points) showed several species but similar genus, then a genus level identification was assigned. However, if the max score (− 5 points) showed multiple species in several genera of the same family, then a family level identification was assigned. In addition, within a 5-point deviation down of the max score, the highest max score and the highest percent identity were also determined. From the top 5 hits down of the max score, an optimized method using the formula, [max score (query cover/identity)], was calculated.

The integrative molecular confirmation combined the simple and optimized BLAST-based sequence matching results with reference of local names, and comparative morphology. As a result, all species identity and generic and familial affinity were further confirmed from the recorded occurrence and distribution of putative species in the study area based on the updated *Co's Digital Flora of the Philippines* [56].

## Results

### Demography of Informants

A total of 335 *Agusan Manobo* key informants (more than 10% of the total *Manobo* population of selected barangays) including traditional healers, leaders, council, and members were interviewed comprised with 106 female and 229 male individuals in an age range from 18–87 years old (median age of 42 years). We considered key informants those who are certified *Agusan Manobo* and knowledgeable with their medicinal plant uses and practices, may it be tribal officials, elders, and members of the community. Demographics by location, educational level, gender, social position, occupation, civil status, and age of participants are summarized in Table 2.

**Table 2:** Sociodemographic profile of the *Manobo* key informants in Sibagat, Esperanza, and Bayugan City, Agusan del Sur

Category	Subcategory	No. of informants	% of informants
Location	Bayugan City	150	44.8
	Sibagat	90	26.9
	Esperanza	95	28.4
Education level	Primary	57	17.0
	Secondary	167	49.9
	Higher education	111	33.1
Gender	Male	229	31.6
	Female	106	68.4
Social Position	Tribal chieftain (Datu)	45	13.4
	Tribal healer	3	0.90
	Tribal IPMR	6	1.80
	Tribal leader	31	9.30
	*Manobo* NCIP focal person	4	1.20
	*Manobo* council of elders	7	2.10
	*Manobo* members	239	71.3
Occupation	Farming	205	61.2
	Animal husbandry	47	14.0
	Employed	49	14.6
	Unemployed	16	4.80
	Others	18	5.40
Civil Status	Single	187	55.8
	Married	133	39.7
	Others	15	4.50
Age	18–34 years old	142	42.4
	35–49 years old	103	30.7
	50–65 years old	53	15.8
	More than 65 years	37	11.0

### Medicinal plant knowledge of *Agusan Manobo*

The majority of the respondents (90.45%) cited their acquisition of medicinal plant knowledge from their parents. They also mentioned other sources of knowledge like fellow tribe band (67.76%), relatives (64.48%), community (61.49%), and through self-discovery (47.76%). However, the descriptive and inferential statistics revealed varying factors affecting the medicinal plant knowledge among the sampled key informants.

When grouped according to location, there was no significant difference on their medicinal plant knowledge as revealed in Kruskal-Wallis test (*p* = 0.379) where the city of Bayugan had the highest number of UR (Md = 112, *n* = 150), followed by the two municipalities, Esperanza (Md = 111, *n* = 95) and Sibagat (Md = 108, *n* = 90). These results showed an exchange of information on these adjacent localities among the *Manobo* community might it be the council of elders and members who are medicinal plant gatherers, peddlers, and traders.

However, when grouped according to education, respondents who had secondary level as their highest educational attainment (Md = 116, *n* = 167) showed the topmost medicinal plant knowledge when compared to primary (Md = 105, *n* = 57) and tertiary (Md = 92, *n* = 111) as revealed by the highly significant difference presented in Kruskal-Wallis test (*p* < 0.001). These results implied that respondents who finished tertiary were more educated with modern medicine and highly acquainted with commercial drugs available over-the-counter for immediate treatment and therapy of their health problems. On the other hand, members with lower educational levels had more medicinal plant knowledge, and most traditional healers, gatherers, and peddlers finished at most on the secondary level.

When grouped according to gender, non-parametric tests revealed that men (Md = 116, *n* = 229) had more medicinal plant knowledge than women (Md = 104, *n* = 106), as demonstrated by the significant difference in both Mann-Whitney *U* test (*p* < 0.001) and Kruskal-Wallis test (*p* < 0.001). It can be observed that men had more medicinal plant knowledge in *Agusan Manobo* culture, an observation supported by the fact that in two of the three selected localities, the tribal healers were males, and most of the tribal officials were also males. These results revealed contrary to the previous statistical findings of [21] in the *Ati* culture of Visayas where women were more knowledgeable than men because they were more involved in medicinal plant gathering and peddling, and women also played a big role in caring for their sick children.

Also, knowledge of the participants when grouped according to social position varied significantly, as revealed by the Kruskal-Wallis test (*p* < 0.001). These results showed that the tribal healers remained the most knowledgeable (Md = 189, *n* = 3), followed by the *Manobo* tribal officials (Md = 172, *n* = 93) with more medicinal plant knowledge when compared to other members of the community (Md = 104, *n* = 239). The medicinal plant knowledge also varied among the *Manobo* tribal officials, namely tribal leaders (Md = 178, *n* = 31), tribal IPMRs (Md = 177, *n* = 6), tribal chieftains (Md = 172, *n* = 45), *Manobo* tribal council of elders (Md = 164, *n* = 7), and *Manobo* NCIP focal persons (Md = 160, *n* = 4).

When grouped according to the occupation, non-parametric Kruskal-Wallis test also significantly revealed (*p* < 0.001) that informants with occupation in farming (Md = 118, *n* = 205) and animal husbandry (Md = 116, *n* = 47) had more medicinal plant knowledge compared to employed (Md = 98, *n* = 49) and unemployed (Md = 96, *n* = 16) informants. These results suggested that *Manobo* people working in line with agriculture were more exposed to medicinal plant knowledge. They were farming crops or raising animals in hinterlands and mountainous areas where most medicinal plants were located. Also, when grouped according to civil status, married informants (Md = 136, *n* = 147) showed higher medicinal plant knowledge than single ones (Md = 92, *n* = 188) as revealed by the very high significant difference in both Mann-Whitney *U* test (*p* < 0.001) and Kruskal-Wallis test (*p* < 0.001). These results implied that married respondents were more exposed during community gatherings, which involved discussions about medicinal plants with regard to their uses and applications. Exchange of information could be observed when couples were present during the scheduled tribal meetings.

Finally, when grouped according to age, descriptive and inferential statistics revealed that respondents from the age group of more than 65 years old had the highest medicinal plant knowledge (Md = 173, *n* = 37), followed by 50–65 years old (Md = 155, *n* = 53), 35–49 years old (Md = 102, *n* = 103), and 18–24 years old (Md = 96, *n* = 142), as revealed by the highly significant difference manifested in Kruskal-Wallis test (*p* < 0.001). These results corresponded to our expectation because older informants most likely had more knowledge of medicinal plant uses and practices based on their long-term experience. These results may also imply that younger generations were becoming more acquainted and educated with modern therapeutic treatment making them more reluctant in their traditional medicinal plant practices like gathering and peddling. This transforming awareness, social, and cultural experiences could influence their medicinal plant interest, traditional knowledge, and attitudes among the *Agusan Manobo*. Younger generations are becoming more privileged to be educated as part of the government scholarship programs for indigenous communities resulting in migration to urban communities.

### Medicinal plants used

A total of 122 reported medicinal plant species belonging to 108 genera and 51 families were classified in 16 use categories, as shown in Tables 3 and 4. All informants interviewed agreed about the healing power of medicinal plants, but only 58.5% of the informants use medicinal plants to treat their health conditions. While some respondents (30.75%) directly relied on seeking for tribal healers in their community, still all these *Babaylans* utilized their known medicinal plants for immediate treatment and therapy. The *Agusan Manobo* community believed that the combined healing gift and prayers of their *Babaylans* could increase the healing potential of their medicinal plants. However, the minority (10.75%) of the key informants depended on seeing a medical practitioner and allied health workers in the treatment of their health conditions at a nearby hospital or health center.

**Table 3:** Use-reports (URs), use values (UVs), and informant consensus factors (ICFs) in every use category (UC).

UC No.	UC names and abbreviations	Reported diseases or uses under each UC	No. of use-report	% of all use-reports	No. of species	% of all species	UV	ICF
1	Diseases caused by bacterial, viral, and parasitic infections (BVP)	Ascariasis, chicken pox, herpes simplex, scabies, jaundice (hepatitis), mumps (parotitis), athlete's foot, warts, amoebiasis, white spot (tinea flava), impetigo, measles, colds (influenza), dengue fever, malaria, typhoid fever, ringworm	3588	8.70	61	9.49	3.04	0.98
2	Tissue growth problems (TGP)	Cancer, cyst, tumor (myoma)	991	2.40	18	2.80	0.95	0.98
3	Endocrine, nutritional, and metabolic (ENM)	Diabetes, tonic, beriberi, hormonal imbalance, goiter	1367	3.31	36	5.60	1.03	0.97
4	Diseases of the nervous system (DNS)	Migraine, Parkinson's disease, nervous breakdown (depression, anxiety, mental stress, nervousness)	239	0.58	7	1.09	0.19	0.97
5	Diseases of the eye (EYE)	Sore eyes, cataract, eye problem (blurred vision, conjunctivitis, eye infection)	308	0.75	8	1.24	0.25	0.98
6	Diseases of the ear (EAR)	Ear congestion, ear infection, discharging ear (otorrhea)	410	0.99	8	1.24	0.36	0.98
7	Diseases of the circulatory system (DCS)	Anemia, hypertension, varicose veins, heart problem (enlargement), internal bleeding, hemorrhage	1333	3.23	31	4.82	0.92	0.98
8	Diseases of the respiratory system (DRS)	Asthma, pneumonia, emphysema, pulmonary tuberculosis, nasal congestion, lung nodule, cough, cough with phlegm, respiratory disease complex (rhinitis, tracheitis, bronchitis), sore throat (tonsillitis)	3896	9.44	67	10.42	2.66	0.98
9	Diseases of the digestive system (DDS)	Constipation, diarrhea, stomach trouble (dysentery, stomachache, bloating), vomiting (nausea), peptic ulcer, toothache, gum swelling, indigestion (dyspepsia), mouth sore (canker sore), stomach acidity (gastritis), swollen/bleeding gums (gingivitis), pancreatitis, liver problem (fatty liver), hemorrhoids, appetite enhancer	6322	15.33	82	12.75	4.64	0.99
10	Diseases of the skin (DOS)	Boils (furuncle/carbuncle), skin eruptions, skin rashes and itchiness (eczema, dermatitis), psoriasis, pimple and acne, hair loss, dandruff	2563	6.21	40	6.22	2.10	0.99
11	Musculoskeletal system and connective tissue problems (MCP)	Joint pain (arthritis, gout), rheumatism, sprain, tendon mass nodule, swollen muscles/swellings, muscle pain	2597	6.30	42	6.53	2.23	0.98
12	Genito-urinary problems (GUP)	Urination difficulty, kidney stones, kidney problem (high uric acid and creatinine), urinary bladder swelling, dysmenorrhea, delayed or irregular menstruation, urinary tract infection	2358	5.72	39	6.07	1.72	0.98
13	Uses in pregnancy to delivery, maternal and infant care (PMI)	Pregnancy (impotence and sterility), abortifacient, labor and delivery enhancer, childbirth tool, miscarriage, maternal care, postpartum care and recovery, new-born baby care, milk production enhancer	1914	4.64	40	6.22	1.25	0.98
14	Abnormal signs and symptoms (ASS)	Abdominal pain, backache, body ache, headache, fever, weakness and fatigue (asthenia), baby teething, child sleeplessness, malaise and fatigue, “pasmo” (cramp and spasm), “bughat” (relapse), skin numbness (paresthesia), dizziness and fainting, body chills, gas pain and flatulence, hangover	8133	19.72	88	13.69	5.84	0.99
15	Other problems of external causes (OEC)	Allergy, burns, cuts and wounds, fracture and dislocation, bruises and contusions, animal bites (snake, dog), insect bites (mosquito, wasp, scorpion), poisoning, contacts with plant or animal parts	5023	12.18	70	10.89	3.98	0.99
16	Other uses (OTU)	Circumcision antiseptic and anesthetic	205	0.50	6	0.93	0.27	0.98

**Table 4:** Medicinal plants used by the *Agusan Manobo* in Agusan del Sur, Philippines

Plant no.	Scientific name	Family	Local name	Voucher no.	UR	UC	UV	CIV	UD	Disease or purpose	Parts used^a^	Preparation and administration^b^		Quantity or dosage	Administration frequency	Experienced adverse or side effects
1	*Andrographis paniculata* Nees	Acanthaceae	White flower	USTH 015616	480	9	1.43	3.07	2.09	Jaundice, colds, malaria; cancer; diabetes; hypertension, heart enlargement, atherosclerosis; cough, respiratory disease complex, sore throat; diarrhea, ulcer, dyspepsia, liver problem; abortifacient; fever, gas pain and flatulence	Lf, Rt	I	Drink decoction	3–5 glasses	Once to thrice a day or as needed	Can cause abortion in pregnant women
										Boils, skin rashes and itchiness, dermatitis	Wh	E	Apply decoction as wash	3–5 glasses	Once a day or as needed	None
2	*Rhinacanthus nasutus* (L.) Kurz	Acanthaceae	Marvelosa or Serpentina	USTH 015622	583	6	1.74	2.90	1.74	Colds; diabetes, beriberi; nervous breakdown; hypertension; diarrhea, stomachache; weakness and fatigue, cramp and spasm	Lf	I	Drink decoction	1–3 glasses	Once a day for 3–5 days	None
3	*Amaranthus spinosus* L.	Amaranthaceae	Kudyapa	USTH 015589	211	9	0.63	2.75	2.06	Diabetes; anemia; cough, bronchitis; dysentery, constipation; urinary tract infection; fever	Lf	I	Drink decoction	3–5 glasses	Thrice a day or as needed	None
										Labor and delivery enhancer	Sd	I	Drink water-infused powdered seeds	1–3 glasses	Twice a day or as needed	None
										Boils, psoriasis, skin rashes, eczema, pimple, acne; snake and scorpion bite	Lf	E	Apply leaves as poultice	3–5 leaves	Thrice a day or as needed	None
4	*Mangifera indica* L.	Anacardiaceae	Mangga	USTH 015591	222	5	0.66	2.85	1.47	Constipation	Fr	I	Eat fresh fruit directly	1–3 fruits	Thrice a day or as needed	None
										Cough, cough with phlegm, sore throat	Lf	I	Drink hot water-infused leaves or decoction	3–5 glasses	Twice a day or as needed	None
										Diarrhea, stomach trouble; headache	Bk	I	Drink decoction	3–5 glasses	Twice a day or as needed	None
										Scabies; cuts and wounds	Bk, Lf	E	Rub crushed leaves or scraped bark	3–5 leaves, 1 palm-sized bark	Twice a day or as needed	None
5	*Spondias pinnata* (L.f.) Kurz	Anacardiaceae	Abihid	USTH 015599	372	4	1.11	2.33	1.39	Colds; diabetes; cough; fever	Bk, Lf	I	Drink decoction of leaves and scraped bark	3–5 glasses	Once or twice a day for 3 days or as needed	None
										Colds; fever	Bk, Lf	E	Bath water-infused leaves and scraped bark	1 pail	Once a day or as needed	None
6	*Annona muricata* L.	Annonaceae	Guyabano	USTH 015593	209	8	0.62	2.17	2.02	Cancer; diabetes; hypertension; dysentery	Fr	I	Eat fresh fruit directly	3–5 glasses	Once or twice a week or as needed	In excess can cause blood viscosity
										Ascariasis; cough; stomach trouble, stomach acidity; urination difficulty, urinary tract infection	Lf	I	Drink decoction	3–5 glasses	Once a day or as needed	None
										Skin eruptions, eczema	Lf, Sp	E	Apply leaf sap or crushed leaves as poultice	3–5 leaves	As needed	None
7	*Cananga odorata* (Lam.) Hook.f. & Thomson	Annonaceae	Anangilan or Ilang-ilang	USTH 015577	358	7	1.07	2.47	1.85	Colds; cough; stomach trouble, ulcer; fever, body chills	Bk, Lf	I	Drink decoction	5–7 glasses	Once or twice a day or as needed	None
										Scabies, athlete's foot; pimple; rheumatism, swollen muscles or swellings, muscle pain; insect bites	Fl	E	Apply oil from steamed flowers	Completely on affected part	3–5 times a day or as needed	None
8	*Friesodielsia lanceolata* (Merr.) Steen.	Annonaceae	Talimughat taas	USTH 015558	198	3	0.59	2.08	0.90	Muscle pain; labor and delivery enhancer, postpartum care and recovery; backache, body ache, weakness and fatigue, cramp and spasm, relapse	Bk, Lf, Rt	I	Drink decoction	3–5 glasses	Once to thrice a day up to 3 days or as needed	None
9	*Uvaria zschokkei* Elmer	Annonaceae	Bigo	USTH 015662	195	5	0.58	0.70	1.56	Amoebiasis; hypertension; fever, weakness and fatigue	St	I	Drink decoction	3–5 glasses	Once to thrice a day or as needed	None
										Hair loss; insect bites	St, Sp	E	Apply stem sap	1/2–1 cup	As needed	None
10	*Alstonia macrophylla* Wall. ex G.Don	Apocynaceae	Dita	USTH 015546	386	9	1.15	2.71	2.04	Tonic; ear congestion; cough; stomach trouble, toothache; urinary tract infection; abdominal pain, weakness and fatigue, hangover	Bk, Lf	I	Drink decoction	3–5 glasses	Once to thrice a day or as needed	None
										Cuts and wounds, bruises and contusions, sprain	Lf	E	Apply crushed and heated leaves as poultice	3–5 leaves	Once or twice a day or as needed	None
										Scabies, impetigo, ringworm; boils	Bk	E	Apply water-infused powdered bark	1 glass	Once or twice a day or as needed	None
										Stomachache, snake bite	Bk	E	Drink local alcohol-tinctured bark	1/2 to 1 glass	As needed	None
11	*Anodendron borneense* (King & Gamble) D.J.Middleton	Apocynaceae	Lunas tag-uli	USTH 015639	1134	12	3.39	3.68	2.22	Cancer; diabetes; ear infections; diarrhea, stomach trouble, ulcer, toothache; arthritis, rheumatism; pregnancy; body ache, weakness and fatigue, cramp and spasm, relapse; poisoning	Sp, St	I	Drink stem sap	1–3 glasses	Once a day or as needed	None
										Colon and prostate cancer, cyst, tumor; diabetes; hypertension; pulmonary tuberculosis; diarrhea, stomach trouble, ulcer, toothache, swollen gums; arthritis, rheumatism; impotence and sterility, postpartum care and recovery; body ache, weakness and fatigue, cramp and spasm, relapse, gas pain, and flatulence; sprain; poisoning	St	I	Drink local alcohol-tinctured or decocted stem	1/2 to 1 glass	Once or twice a day or as needed	None
										Scabies, warts, impetigo, typhoid fever; boils, skin eruptions, skin rashes, and itchiness; arthritis, rheumatism, swellings, muscle pain; backache, body ache, weakness and fatigue, cramp and spasm, relapse gas pain and flatulence; allergy, burns, cuts and wounds, sprain, animal and insect bites, contacts with plants and animal parts	St	E	Apply coconut or Efficascent oil-infused stem	Completely on affected part	Once or twice a day or as needed	None
12	*Hoya imbricata* Decne.	Apocynaceae	Pikot-pikot	USTH 015618	57	2	0.17	0.86	0.69	Boils; cuts and wounds	Lf	E	Apply coconut oil-infused burned and powdered leaves	Completely on affected part	As needed	None
13	*Alocasia zebrina* Schott ex Van Houtte	Araceae	Lunas gabi	USTH 015614	44	1	0.13	0.60	0.00	Allergy, cuts and wounds, snake and insect bite, poisoning	Lf, Sp, St	E	Apply stem or leaf sap	Completely on affected part	Once a day or as needed	None
14	*Homalomena philippinensis* Engl. ex Engl. & K.Krause	Araceae	Payaw	USTH 015597	466	7	1.39	2.00	1.83	Colds; body ache, headache, fever	Lf, St	I/E	Sniff sliced and pounded leaf and stem or tie leaf and stem around the neck	1–3 leaves	Once a day or as needed	None
										Tonsillitis; pregnancy, impotence and sterility, labor and delivery enhancer	Rz	I	Drink extracted juice from crushed rhizome	1–3 cups	Once to thrice a day or as needed	None
										Rheumatism; cuts and wounds	Rz	E	Apply extracted juice from crushed rhizome	Completely on affected part	As needed	None
										Hemorrhoids	Lf	E	Insert heated young leaf	1 leaf	Once or twice a day or as needed	None
15	*Hydrocotyle vulgaris* L.	Araliaceae	Goto Kola	USTH 015563	263	4	0.78	1.78	1.39	Diabetes; hypertension; fever	Lf	I	Eat fresh leaves directly or drink decocted leaves	3–5 leaves; 1 cup	Once a day or as needed	In excess can cause anemia, dizziness and weakening
										Cuts and wounds	Lf, Sp	E	Apply leaf sap or crushed leaves as poultice	1–3 leaves	As needed	None
16	*Areca catechu* L.	Arecaceae	Huling-huling	USTH 015610	42	1	0.13	0.70	0.69	Breast cancer	Rt	I	Drink decoction	3–5 glasses	Once or twice a day or as needed	None
17	*Calamus megaphyllus* Becc.	Arecaceae	Kapi	USTH 015608	168	4	0.50	1.65	1.28	Hypertension; asthma; diarrhea, dyspepsia, gastritis, indigestion; arthritis, rheumatism	Rz	I	Drink decoction	3–5 glasses	Twice a day or as needed	None
18	*Thottea affinis* (Planch. ex Rolfe) ined.	Aristolochiaceae	Salimbagat	USTH 015643	278	3	0.83	1.75	1.10	Amoebiasis; cancer; toothache	Lf, Rt	I	Drink decoction	3–5 glasses	Once a day or as needed	None
19	*Dracaena roxburghiana* (Schult.f.) Byng & Christenh.	Asparagaceae	Espada-espada	USTH 015647	78	2	0.23	0.67	0.69	Boils; snake bite	Lf	E	Apply leaf sap or pounded leaves as poultice	5–7 drops	As needed	None
20	*Acmella grandiflora* (Turcz.) R.K.Jansen	Asteraceae	Lunas pilipo	USTH 015548	396	4	1.18	2.40	1.33	Toothache; anesthetic	Fl	I	Apply fresh flower directly	1–3 flowers	As needed	None
										Skin rashes and itchiness, psoriasis; cuts and wounds; anesthetic	Fl, Lf	E	Apply crushed flower or leaves as poultice	1–3 flowers, 5–7 leaves	As needed	None
21	*Ageratum conyzoides* L.	Asteraceae	Albahaca	USTH 015602	77	3	0.23	1.89	1.10	Abortifacient; weakness and fatigue	Lf	I	Drink decoction	1–3 cups	Once a day or as needed	Can cause abortion in pregnant women
										Cuts and wounds		E	Apply pounded leaves as poultice	1–3 leaves	Once or twice a day or as needed	None
22	*Artemisia vulgaris* L.	Asteraceae	Helbas	USTH 015619	365	4	1.09	1.60	1.24	Asthma, cough, cough with phlegm; diarrhea, dyspepsia; delayed menstruation; relapse	Lf	I	Drink decoction	1–3 glasses	Thrice a day or as needed	In excess can cause anemia, dizziness and weakening
										Abdominal pain, body ache, fever, cramp, and spasm	Lf	E	Apply crushed leaves as poultice	3–5 leaves	Once a day or as needed	None
23	*Bidens pilosa* L.	Asteraceae	Tuway-tuway	USTH 015582	218	5	0.65	1.67	1.26	Colds; diarrhea; muscle pain; backache, body ache, fever, weakness and fatigue, cramp and spasm, relapse, gas pain, and flatulence	Rt	I	Drink decoction	3–5 glasses	Once or twice a day up to 3 days or as needed	None
										Cuts and wounds, animal and insect bites	Lf	E	Apply crushed leaves as poultice	3–5 leaves	Once to thrice a day or as needed	None
24	*Blumea balsamifera* (L.) DC.	Asteraceae	Gabon	USTH 015573	412	6	1.23	2.60	1.58	Hypertension; cough, cough with phlegm; urination difficulty; postpartum care and recovery; body ache, headache, fever, weakness and fatigue, gas pain and flatulence	Lf, Rt	I	Drink decoction	3–5 glasses	Once or twice a day for 3 days or as needed	None
										Headache	Lf	E	Apply steamed or pounded leaves in the forehead	1–3 leaves	Once a day or as needed	None
										Boils, skin rashes	Lf	E	Apply leaves as poultice	1–3 leaves	Once or twice a day or as needed	None
25	*Chromolaena odorata* (L.) R.M.King & H.Rob.	Asteraceae	Hagonoy	USTH 015632	448	5	1.34	2.50	1.56	Tumor; hemorrhage; fever	Lf	I	Drink decoction	3–5 glasses	Once a day for 3 days or as needed	None
										Boils; burns, cuts, and wounds	Lf	E	Apply leaf sap or crushed leaves as poultice	3–5 leaves	As needed	None
26	*Cyanthillium cinereum* (L.) H.Rob.	Asteraceae	Kanding-kanding	USTH 015587	476	5	1.42	2.78	1.42	Colds, malaria; pulmonary tuberculosis; dog bite	Lf, Rt	I	Drink decoction	3–5 glasses	Thrice a day or as needed	None
										Chicken pox, herpes simplex, measles; boils, skin eruptions, skin rashes and itchiness; weakness and fatigue, cramp and spasm	Fl, Lf, Rt	E	Bath water-infused leaves and roots or burn leaves and roots as incense	1 pail as bath or 1 bowl as incense	Once or twice a day or as needed	None
27	*Erechtites valerianifolius* (Link ex Spreng.) DC.	Asteraceae	Gapas-gapas bae	USTH 015666	208	3	0.62	2.25	1.01	Stomachache, dyspepsia; body ache, headache, gas pain, and flatulence	Lf	I	Drink decoction	3–5 glasses	Once or twice a day or as needed	None
										Cuts and wounds	Lf, Sp	E	Apply sap or leaves as poultice	3–5 leaves	As needed	None
28	*Gynura procumbens* (Lour.) Merr.	Asteraceae	Ashitaba	USTH 015645	215	4	0.64	2.50	1.33	Emphysema, cough; diarrhea, stomach trouble; kidney stones; abdominal pain	Lf	I	Drink brewed tea-prepared leaves or decoction	3–5 cups	Once or twice a day or as needed	None
29	*Mikania cordata* (Burm.f.) B.L.Rob.	Asteraceae	Moti-moti	USTH 015543	397	6	1.19	2.75	1.67	Cough; ulcer	Lf	I	Drink decoction	3–5 glasses	Twice a day or as needed	None
										Sore eyes	Lf, Sp	I	Drop leaf sap	Completely on affected part	As needed	None
										Skin rashes and itchiness; cuts and wounds, snake and scorpion bites; circumcision antiseptic	Lf	E	Apply leaf sap or crushed leaves as poultice	5–7 leaves	As needed	None
30	*Pseudelephantopus spicatus* (Juss.) Rohr	Asteraceae	Kukog banog	USTH 015564	500	5	1.49	2.50	1.44	Urination difficulty, kidney problem, urinary bladder swelling, delayed menstruation, urinary tract infection; fever, weakness and fatigue, cramp and spasm	Lf, Rt	I	Drink brewed tea-prepared leaves or decoction	3–5 glasses	Once a day or as needed	None
										Sore eyes; eczema, skin rashes, and itchiness; cuts and wounds, sprain, snake bite	Lf, Sp	E	Apply drops of leaf sap	Completely on affected part	Thrice a day or as needed	None
31	*Diplazium esculentum* (Retz.) Sw.	Athyriaceae	Pako-pako	USTH 015545	212	5	0.63	1.92	1.56	Colds; cough; diarrhea, dysentery; labor and delivery enhancer, postpartum care and recovery; body ache, headache, fever	Sh	I	Drink decoction	3–5 glasses	Twice a day or as needed	None
32	*Begonia contracta* Warb.	Begoniaceae	Budag-budag	USTH 015654	85	2	0.25	1.33	0.64	Pimple, dandruff; burns	Fl, Lf	E	Apply crushed flower and leaves as poultice	1–3 flowers, 1–3 leaves	Once to thrice a day or as needed	None
33	*Ceiba pentandra* (L.) Gaertn.	Bombacaceae	Doldol	USTH 015535	140	5	0.42	2.14	1.55	Diabetes; pulmonary tuberculosis; diarrhea, dysentery; rheumatism, swollen muscles; snake bite	Bk, Rt	I	Drink decoction	3–5 glasses	Once to thrice a day or as needed	None
34	*Ehretia microphylla* Lam.	Boraginaceae	Alangitngit or Tsaang-Gubat	USTH 015638	336	4	1.00	2.60	1.39	Diabetes; nervous breakdown; stomach acidity; food and drug allergy	Lf	I	Drink tea-prepared leaves	1/2 to 1 cup	Once a day for 3 days or as needed	None
35	*Ananas comosus* (L.) Merr.	Bromeliaceae	Pinya	USTH 015667	226	7	0.67	1.71	1.85	Ascariasis, amoebiasis; cancer; diabetes; hypertension; constipation, stomach acidity	Fr	I	Eat fresh fruit directly	1–3 slices	Once to thrice a day or as needed	None
										Headache, fever, weakness, and fatigue	Lf, Sh	E	Apply crushed shoot or leaves as poultice	Completely on affected part	As needed	None
										Cancer; swellings	Lf	I/E	Drink decoction or apply decocted leaves	3–5 leaves	Once a day or as needed	None
36	*Abroma augusta* (L.) L.f.	Byttneriaceae	Samboligawn	USTH 015637	329	8	0.98	2.69	1.98	Diabetes, tonic; bronchitis; stomachache; dysmenorrhea, irregular menstruation; sterility	Bk, Lf, Rt	I	Drink decoction	3–5 glasses	Once a day or as needed	None
										Scabies; boils, skin eruptions, dermatitis; cuts and wounds	Bk, Lf	E	Apply decoction as wash	1–3 glasses	Once or twice a day or as needed	None
37	*Kleinhovia hospita* L.	Byttneriaceae	Bitan-ag	USTH 015631	146	6	0.44	2.50	1.70	Tumor; asthma, pneumonia, cough; dyspepsia, liver problem; headache; baby teething	Lf	I	Drink decoction	3–5 glasses	Thrice a day or as needed	None
										Scabies; psoriasis	Lf	E	Apply crushed leaves as poultice	3–5 leaves	Once to thrice a day or as needed	None
38	*Melochia umbellata* (Houtt.) Stapf	Byttneriaceae	Banitlong	USTH 015649	265	4	0.79	1.76	1.24	Rheumatism; backache, body ache, headache	Lf	I	Drink decoction	3–5 glasses	Once to thrice a day or as needed	None
										Canker sore; burns	Lf	E	Apply leaves as poultice	3–5 leaves	Once a day or as needed	None
39	*Hippobroma longiflora* (L.) G.Don	Campanulaceae	Elepanteng puti	USTH 015583	213	5	0.64	1.83	1.56	Toothache	Lf	I	Apply chewed or pounded leaves	1–3 leaves	Once or twice a day or as needed	None
										Nervous breakdown; asthma, bronchitis; fever	Lf	I	Drink decoction	3–5 glasses	Once to thrice a day or as needed	None
										Cuts and wounds	Lf	E	Apply decoction	1 glass	As needed	None
40	*Carica papaya* L.	Caricaceae	Kapayas laki	USTH 015668	659	6	1.97	2.92	1.64	Constipation, dyspepsia; milk production enhancer	Fr	I	Eat fresh fruit directly	1–3 slices	Once a day or as needed	None
										Tonic; asthma; stomach problem	Lf, Rt	I	Drink decoction	3–5 glasses	Once a day or as needed	None
										Dengue fever	Lf, Sp	I	Drink leaf sap	5–7 leaves	Thrice a day or as needed	None
										Body ache, fever, cramp, and spasm	Lf	I	Apply crushed and heated leaves as poultice	1–3 leaves	Twice a day or as needed	None
41	*Cratoxylum sumatranum* (Jack) Blume	Clusiaceae/Guttiferae	Bansilay	USTH 015541	96	4	0.29	2.33	1.33	Colds; cough; dysentery	Bk, Lf, Rt	I	Drink decoction	3–5 glasses	Once to thrice a day or as needed	None
										Toothache	Lf	I	Apply chewed or pounded leaves	3–5 leaves	Once to thrice a day or as needed	None
										Impetigo; cuts and wounds	Lf	E	Apply pounded leaves as poultice	3–5 leaves	Once to thrice a day or as needed	None
42	*Hellenia speciosa* (J.Koenig) Govaerts	Costaceae	Tambabasi or Tawasi	USTH 015578	744	8	2.22	2.58	2.03	Diabetes, goiter; migraine; ear congestion; cough, lung nodule; urination difficulty, kidney problem; headache, fever	Lf, Rz	I	Drink decoction	3–5 glasses	Once to thrice a day up to 3 days or as needed	None
										Diarrhea, stomachache, dysentery	St	I	Drink stem sap	1/2 cup	As needed	None
										Sore eyes	Lf	I	Apply leaf sap	Completely on affected part	As needed	None
43	*Kalanchoe pinnata* (Lam.) Pers.	Crassulaceae	Hanlilika	USTH 015584	486	12	1.45	2.88	2.21	Diabetes; anemia, hypertension; asthma; cough; constipation, diarrhea, stomach trouble, hemorrhoids; kidney stone; labor and delivery enhancer; fever	Lf	I	Drink decoction	3–5 leaves	Once a day or as needed	None
										Herpes simplex; hemorrhoids; boils, eczema; swellings; burns, cuts and wounds, bruises and contusions, insect bites	Lf	I	Apply decocted leaves as wash	1–3 leaves	Once a day or as needed	None
										Abdominal pain, body ache, headache, fever	Lf	E	Apply heated leaves as hot compress	1–3 leaves	Once a day or as needed	None
44	*Rhynchospora colorata* (L.) H.Pfeiff.	Cyperaceae	Busikad	USTH 015571	254	6	0.76	1.38	1.61	Chicken pox, measles; cancer; cough; stomach acidity; fever, relapse, gas pain and flatulence; sprain	Wh	I	Drink decoction	1–3 glasses	Once to thrice a day or as needed	None
										Baby teething	Fl	I	Drink water-infused flower	1/2–1 glass	Once to thrice a day	None
45	*Stenomeris borneensis* Oliv.	Dioscoreaceae	Banag	USTH 015537	540	6	1.61	2.36	1.70	Myoma; migraine; arthritis, rheumatism; urination difficulty, urinary bladder swelling; postpartum care and recovery; headache, cramp and spasm, relapse	Rt	I	Drink decoction	3–5 glasses	Once or twice a day for 3 days or as needed	None
46	*Euphorbia hirta* L.	Euphorbiaceae	Tawa-tawa	USTH 015665	305	7	0.91	2.80	1.85	Colds, dengue fever; asthma; diarrhea, vomiting; fever	Wh	I	Drink decoction of whole plant except flowers	5–7 glasses	Thrice a day or as needed	In excess can cause thrombocytopenia
										Ringworm; sore eyes; boils, skin rashes, and itchiness; cuts and wounds	Lf	I/E	Apply leaf sap or decocted leaves	5–7 leaves	Thrice a day or as needed	None
47	*Jatropha curcas* L.	Euphorbiaceae	Tuba-tuba puti	USTH 015595	495	7	1.48	2.66	1.79	Colds; pulmonary tuberculosis; diarrhea; arthritis, rheumatism; backache, body ache, fever, weakness and fatigue, cramp and spasm, relapse, gas pain, and flatulence	Lf, Rt	I	Drink decoction	1–3 leaves	Once a day or as needed	None
										Scabies, ringworm; ear infection, discharging ear; toothache; swollen muscles and swellings; cuts and wounds, fracture and dislocation, animal and insect bites	Bk, Rt	I/E	Apply decoction or pounded scraped bark as poultice	1–3 palm-sized barks, 1/2–1 arm-sized roots	As needed	None
48	*Jatropha gossypifolia* L.	Euphorbiaceae	Tuba-tuba tapol	USTH 015586	810	9	2.41	2.83	1.94	Colds, malaria, typhoid fever; pulmonary tuberculosis; diarrhea; arthritis, rheumatism; dysmenorrhea, irregular menstruation; backache, body ache, fever, weakness and fatigue, cramp and spasm, relapse, gas pain, and flatulence	Lf, Rt	I	Drink decoction	1–3 leaves, 1/2–1 arm-sized roots	Once a day or as needed	None
										Ringworm; boils, carbuncles, dermatitis; swollen muscles and swellings, muscle pain; backache, body ache, fever; cuts and wounds	Lf	E	Bath or wash decocted leaves	1–3 leaves	Once a day or as needed	None
										Scabies, ringworm; ear infection, discharging ear; toothache, mouth sore; cuts and wounds, fracture and dislocation, animal and insect bites	Bk, Rt	I/E	Apply decoction or pounded scraped bark as poultice	1–3 palm-sized barks, 1/2–1 arm-sized roots	As needed	None
49	*Melanolepis multiglandulosa* (Reinw. ex Blume) Rchb. & Zoll.	Euphorbiaceae	Awom	USTH 015621	485	5	1.45	2.33	1.56	Beriberi; emphysema, cough; diarrhea, stomach trouble	Lf	I	Drink decoction	3–5 glasses	Once to thrice a day or as needed	None
										Fibroma; body ache, weakness, and fatigue	Bk, Fl, Lf	E	Apply fresh or heated flower, leaves, and bark; sometimes mixed with little salt	1–3 flowers, 1–3 leaves, 1–3 palm-sized barks	Once or twice a day or as needed	None
50.1	*Omalanthus macradenius* Pax & Hoffm.	Euphorbiaceae	Banti puti	USTH 015633	202	3	0.60	1.77	1.04	Impetigo; diarrhea, stomach trouble; cuts and wounds	Lf	E	Apply pounded leaves as poultice	3–5 leaves	Once to thrice a day or as needed	None
50.2	*Omalanthus macradenius* Pax & Hoffm.	Euphorbiaceae	Banti tapol	USTH 015554	203	3	0.61	1.60	1.04	Impetigo; diarrhea, stomach trouble; cuts and wounds	Lf	E	Apply pounded leaves as poultice	3–5 leaves	Once to thrice a day or as needed	None
51	*Bauhinia* sp.	Fabaceae	Talimughat pikas	USTH 015575	284	4	0.85	1.50	1.22	Rheumatism, muscle pain; delayed menstruation; labor and delivery enhancer, postpartum care and recovery; backache, body ache, weakness and fatigue, cramp and spasm, relapse	Lf, St	I	Drink decoction	3–5 glasses	Once to thrice a day up to 3 days or as needed	None
52	*Crotalaria incana* L.	Fabaceae	Sagay-sagay	USTH 015572	84	5	0.25	1.60	1.24	Myoma; hormonal imbalance; cough; constipation; fever, weakness and fatigue, relapse	Rt	I	Drink decoction	3–5 glasses	Once to thrice a day up to 3 days or as needed	None
53	*Gliricidia sepium* (Jacq.) Kunth ex Steud*.*	Fabaceae	Madre de Cacao	USTH 015620	153	6	0.46	1.83	1.68	Scabies; boils, skin eruption, skin rashes, and itchiness; cuts and wounds	Lf, Sp	E	Apply leaf sap or pounded leaves as poultice	Completely on affected part	Once or twice a day or as needed	None
										Eczema, dermatitis; arthritis and rheumatism; burns, cuts and wounds, bruises and contusions	Bk, Rt, Sp	E	Apply sap or decocted bark or root	Completely on affected part	Once or twice a day or as needed	None
										Abortifacient, postpartum care, and recovery	Lf	E	Burn leaves as incense or apply heated leaves as hot compress	3–5 leaves	Once a day or as needed	None
										Body ache, headache, fever; fracture and dislocation, sprain	Bk	E	Apply scraped bark as poultice	1–3 palm-sized barks	Once a day or as needed	None
54	*Mimosa pudica* L.	Fabaceae	Hibi-hibi or makahiya	USTH 015570	355	8	1.06	2.29	1.97	Diabetes; hypertension; asthma, dysentery; urination difficulty; fever	Rt	I	Drink decoction	3–5 glasses	Once a day or as needed	None
										Baby teething	Rt	I	Drink water-infused peeled roots	1/2 to 1 cup	Once a day or as needed	None
										Mumps; boils; child sleeplessness, malaise, and fatigue	Sh	E	Apply hot water-infused shoots	1/2 to 1 glass	As needed	None
55	*Ormosia macrodisca* Baker	Fabaceae	Bahay	USTH 015625	522	5	1.56	2.36	1.56	Atherosclerosis (high cholesterol)	Fr	I	Eat fresh fruit directly	1–3 fruits	Once or twice a day or as needed	None
										Typhoid fever; nervous breakdown; high cholesterol; kidney problem; fever	Bk	I	Drink decoction or local alcohol-tinctured bark	1/2 to 1 cup	Once or twice a day or as needed	None
										Nervousness; skin numbness	Bk, Rt	E	Apply Efficascent oil-infused bark and root	Fill a 250 ml glass bottle with bark and roots	As needed	None
56.1	*Phanera semibifida* (Roxb.) Benth.	Fabaceae	Alibangbang puti	USTH 015646	66	1	0.20	1.11	0.00	Internal bleeding, hemorrhage	Lf	I	Drink decoction	3–5 glasses	Once to thrice a day or as needed	None
56.2	*Phanera semibifida* (Roxb.) Benth.	Fabaceae	Alibangbang tapol	USTH 015634	53	1	0.16	1.00	0.00	Internal bleeding, hemorrhage	Lf	I	Drink decoction	3–5 glasses	Once to thrice a day or as needed	None
57	*Callicarpa pedunculata* R.Br.	Lamiaceae	Awoy	USTH 015661	378	4	1.13	1.50	1.28	Ulcer, pancreatitis, fatty liver; weakness and fatigue, cramp and spasm	Lf	I	Drink hot water-infused leaves	1/2 to 1 cup	Once or twice a day or as needed	None
										Asthma	Lf	E	Burn leaves as incense	1–3 leaves	Once or twice a day or as needed	None
										Swollen muscles, muscle pain; backache, body ache	Lf	E	Apply leaves as poultice	1–3 leaves	Once or twice a day or as needed	None
58	*Coleus amboinicus* Lour.	Lamiaceae	Kalabo	USTH 015617	380	4	1.13	1.78	1.31	Asthma, cough, cough with phlegm; dyspepsia; abdominal pain, gas pain, and flatulence	Lf	I	Eat leaves directly or drink decoction	1/2 to 1 cup	Once to thrice a day or as needed	In excess can cause anemia, weakness, and allergy
										Burns, bruised and contusions, insect bites	Lf	E	Apply water-infused leaves	1–3 glasses	As needed	None
59.1	*Coleus scutellarioides* (L.) Benth.	Lamiaceae	Mayana kanapkap	USTH 015567	260	5	0.78	1.67	1.47	Anemia; asthma, pneumonia, cough; dyspepsia; gas pain and flatulence	Lf	I	Drink decoction	1–3 glasses	Once a day for 3–5 days or as needed	None
										Cuts and wounds, bruises and contusions, sprain	Lf, Sp	E	Apply leaf sap or crushed leaves as poultice	5–7 leaves	Twice a day or as needed	None
59.2	*Coleus scutellarioides* (L.) Benth.	Lamiaceae	Mayana pula	USTH 015644	414	6	1.24	2.25	1.59	Anemia; asthma, pneumonia, emphysema, pulmonary tuberculosis, cough; ulcer, dyspepsia; gas pain and flatulence	Lf	I	Drink decoction	1–3 glasses	Once a day for 3–5 days or as needed	None
										Conjunctivitis	Lf	I	Apply decoction as drop	Completely on affected part	Once or twice a day or as needed	None
										Cuts and wounds, bruises and contusions, sprain	Lf	E	Apply crushed leaves as poultice	5–7 leaves	Twice a day or as needed	None
60	*Gmelina arborea* Roxb. ex Sm.	Lamiaceae	Gmelina	USTH 015635	335	5	1.00	1.83	1.49	Toothache, gum swelling	Lf	I	Apply chewed or pounded leaves	3–5 leaves	As needed	None
										Discharging ear	Fr	I	Drop extract of heated fruit	1–3 fruits	As needed	Poisonous when eaten
										Stomach bloating; maternal care; headache, gas pain and flatulence; cuts and wounds	Lf	E	Apply leaves directly or as poultice	1–3 leaves	As needed	None
61	*Hyptis capitata* Jacq.	Lamiaceae	Sawan-sawan	USTH 015574	498	7	1.49	2.56	1.85	Colds, malaria; cough; diarrhea, stomachache; new-born baby care; fever, gas pain and flatulence	Lf	I	Drink decoction or leaf sap	3–5 glasses decoction or 1/2 cup leaf sap (adult); 1/2 cup decoction or 1 teaspoonful leaf sap (baby)	Once or twice a day or as needed	None
										Delayed menstruation	Rt	I	Drink decoction	3–5 glasses	Once to thrice a day or as needed	None
										Toothache; cuts and wounds	Lf	E	Apply crushed leaves as poultice	3–5 leaves	As needed	None
62	*Mentha arvensis* L.	Lamiaceae	Herba buena	USTH 015669	174	6	0.52	2.71	1.59	Measles; cough; diarrhea, dysentery; dysmenorrhea; headache, fever, cramp and spasm, gas pain and flatulence	Sh	I	Drink decoction	3–5 glasses	Once or twice a day or as needed	None
										Asthma; dizziness and fainting	Lf	I	Sniff crushed leaves or leaves infused with hot water	3–5 leaves	As needed	None
										Toothache; headache, fever; insect bites	Lf	E	Apply chewed or crushed leaves	3–5 leaves	As needed	None
63	*Mentha canadensis* L.	Lamiaceae	Sencia	USTH 015670	432	9	1.29	2.81	2.04	Sinusitis, cough; stomachache, vomiting; delayed menstruation; backache, body ache, headache, fever, gas pain and flatulence	Lf	I	Drink hot water-infused leaves or decoction	3–5 glasses	Once or twice a day or as needed	None
										Ringworm; ear infection and congestion; toothache	Lf	I/E	Apply leaf sap	Completely on affected part	As needed	None
										Muscle pain, abdominal pain; cuts and wounds, dislocation, snake bite	Lf	E	Apply crushed leaves as poultice	Completely on affected part	Once a day or as needed	None
										Skin rashes and itchiness, acne; rheumatism; cuts and wounds; animal and insect bites	Lf	E	Apply decoction as wash	5–7 leaves	Twice a day or as needed	None
64	*Ocimum basilicum* L.	Lamiaceae	Sangig	USTH 015630	385	9	1.15	2.33	2.09	Cough, cough with phlegm; constipation, diarrhea, vomiting, hemorrhoids; delayed menstruation; postpartum care and recovery; headache, fever, gas pain and flatulence	Lf, Sh	I	Drink decoction or add in soup	3–5 glasses	Twice a day or as needed	None
										Ear congestion, infection, and discharge	Lf, Sp	I	Drop leaf sap	3–5 leaves	As needed	None
										Boils, skin rashes, and itchiness; arthritis, rheumatism; cuts and wounds, bruises and contusions	Lf	E	Apply decoction as wash	3–5 leaves	Twice a day or as needed	None
										Toothache; cuts and wounds, snake bites	Lf, Sh	I/E	Apply crushed shoot or leaves as poultice	3–5 leaves, 1 shoot	As needed	None
65	*Orthosiphon aristatus* (Blume) Miq.	Lamiaceae	Wachichao	USTH 015550	513	6	1.53	2.96	1.58	Diabetes; hypertension; diarrhea, stomachache; joint pain, gout, rheumatism; urination difficulty, kidney stones, kidney problem, urinary bladder swelling, prostate problem; labor and delivery enhancer	Fl, Lf	I	Drink brewed tea-prepared leaves or decoction of leaves and flower	3–5 cups	Once or twice a day or as needed	None
66	*Premna odorata* Blanco	Lamiaceae	Abgaw	USTH 015559	668	7	1.99	2.94	1.79	Colds; nasal congestion, sinusitis, cough, cough with phlegm; diarrhea, ulcer; rheumatism; postpartum care and recovery; weakness and fatigue, gas pain and flatulence	Lf	I	Drink water-infused leaves	3–5 glasses	Once or twice a day for 3 days or as needed	None
										Cuts and wounds		E	Apply crushed leaves as poultice	1–3 leaves	Once or twice a day or as needed	None
67	*Teijsmanniodendron ahernianum* (Merr.) Bakh.	Lamiaceae	Kulipapa	USTH 015603	128	4	0.38	1.18	1.24	Beriberi; muscle pain; labor and delivery; backache, body ache, cramp and spasm	Rt, St	I	Drink decoction	3–5 glasses	Thrice a day or as needed	None
68	*Vitex negundo* L.	Lamiaceae	Lagundi	USTH 015562	475	5	1.42	2.69	1.55	Cough, cough with phlegm; ulcer; rheumatism; postpartum care and recovery; headache, gas pain and flatulence	Lf	I	Drink decoction	1/4 glass (young leaf) or 1/2 glass (mature leaf)	Thrice a day or as needed	None
69	*Cinnamomum mercadoi* S.Vidal	Lauraceae	Kaningag	USTH 015585	908	8	2.71	3.22	1.93	Amoebiasis; cancer; hypertension; cough; diarrhea, stomach trouble, ulcer, stomach acidity; kidney problem, urinary tract infection; weakness and fatigue, cramp and spasm	Bk, Br, Rt	I	Drink decoction or local alcohol-tinctured bark, stem and root	3–5 glasses	Once or twice a day or as needed	None
										Cuts and wounds	Bk, Br, Rt	E	Apply coconut oil-infused bark, stem and root	Completely on affected part	As needed	None
70	*Litsea cordata* (Jack) Hook.f.	Lauraceae	Loktob	USTH 015580	307	7	0.92	2.83	1.79	Mumps; cyst, tumor, myoma; goiter; asthma, pneumonia, emphysema, cough; ulcer; arthritis; kidney problem, dysmenorrhea	Bk, Rt	I	Drink hot water-infused bark or decoction	1–3 glasses	Once a day in thrice a week for 2 months or as needed	In excess can cause anemia, dizziness and weakening
71	*Machilus philippinensis* Merr.	Lauraceae	Efficascent	USTH 015576	82	2	0.24	1.11	0.69	Cough; weakness and fatigue	Sp, St	I	Drink sap from rubbed stem	1/2 cup	Once a day or as needed	None
72	*Lagerstroemia speciosa* (L.) Pers.	Lythraceae	Banaba	USTH 015596	384	4	1.15	2.57	1.26	Ulcer; urination difficulty, kidney stones, high uric acid, and creatinine; maternal care; backache, body ache, fever	Lf	I	Drink decoction	3–5 glasses	Once to thrice a day or as needed	None
73	*Gossypium hirsutum* L.	Malvaceae	Gapas	USTH 015553	283	3	0.84	2.14	0.95	Hemorrhage; postpartum care and recovery; body ache, fever, body chills	Rt	I	Drink decoction	1 glass	Once a day for 3 days	In excess, can cause abnormalities in lactating mothers
74	*Sida rhombifolia* L.	Malvaceae	Eskuba laki	USTH 015601	768	8	2.29	2.55	1.87	Cough; stomach trouble; kidney stone, kidney problem, prostate problem, irregular menstruation	Lf, Rt	I	Drink decoction	3–5 glasses	Once a day or as needed	None
										Chicken pox, herpes simplex, scabies; boils; swellings; backache, body ache, headache; cuts and wounds	Lf, Rt	E	Apply leaves as poultice or leaf and bark decoction as wash	3–5 leaves	As needed	None
										Fever	Bk	I	Drink decoction	1–3 palm-sized barks	Once or twice a day or as needed	None
75	*Urena lobata* L.	Malvaceae	Dupang bae	USTH 015664	482	7	1.44	2.06	1.80	Stomach trouble; arthritis, rheumatism; labor and delivery, postpartum care and recovery; fever; cuts and wounds, fracture and dislocation, bruises and contusion, sprain, animal bites	Wh	I/E	Drink or apply decoction or burn as incense	1 bowl	Once a day or as needed	None
										Diabetes; sore throat; toothache; abdominal pain	Sh	I	Drink decoction	3–5 glasses	Once or twice a day or as needed	None
76	*Angiopteris evecta* Sw.	Marattiaceae	Amampang	USTH 015658	126	3	0.38	1.50	0.87	Muscle pain; postpartum care and recovery; backache, body ache, weakness, and fatigue, cramp and spasm	Rt	I	Drink decoction	3–5 glasses	Once to thrice a day or as needed	None
77	*Medinilla teysmannii* Miq.	Melastomataceae	Tampion	USTH 015581	282	3	0.84	1.25	1.04	Swollen muscles and swellings, muscle pain; gas pain and flatulence; sprain	Lf	E	Apply heated leaves as hot compress	1–3 leaves	Once a day or as needed	None
78	*Melastoma malabathricum* L.	Melastomataceae	Hantutuknaw puti	USTH 015588	274	3	0.82	1.89	0.96	Diarrhea, dysentery, stomachache, hemorrhoids; headache, fever	Sh	I	Drink decoction	3–5 glasses	Once a day or as needed	None
										Toothache; cuts and wounds	Lf	I/E	Drop or drink stem sap	1–3 leaves	As needed	None
79	*Lansium domesticum* Correa	Meliaceae	Lansones	USTH 015565	103	4	0.31	1.52	1.28	Malaria; diarrhea, dysentery, dyspepsia; fever, gas pain and flatulence	Bk, Lf	I	Drink decoction	3–5 glasses	Twice a day or as needed	None
										Insect bites	Bk	E	Apply powdered bark	Completely on affected part	As needed	None
80	*Sandoricum koetjape* (Burm.f.) Merr.	Meliaceae	Santol	USTH 015624	464	7	1.39	1.78	1.85	Tonic; hypertension; diarrhea, dysentery; postpartum care and recovery; abdominal pain, fever	Bk, Fr, Lf	I	Drink decoction of mesocarp, leaves and scraped bark	3–5 glasses	Once a day or as needed	None
										Toothache	Lf	I	Apply crushed leaves as poultice	1–3 leaves	As needed	None
										Boils, skin rashes and itchiness, dermatitis	Lf	E	Apply decoction as wash	3–5 leaves	Once or twice a day or as needed	None
										Ringworm	Bk	E	Apply pounded scraped bark as poultice	1–3 palm-sized barks	Once or twice a day or as needed	None
81	*Swietenia mahagoni* (L.) Jacq.	Meliaceae	Mahogany	USTH 015671	334	9	1.00	2.29	2.14	Dysmenorrhea, delayed menstruation; abortifacient; abdominal pain	Sd	I	Take powdered seed or drink decoction	1–3 glasses	Once a day or as needed	Can cause abortion in pregnant women
										Amoebiasis, malaria; cancer; tonic; hypertension; cough; diarrhea; miscarriage; fever	Bk	I	Drink decoction	1–3 glasses	Once a day or as needed	None
82	*Arcangelisia flava* (L.) Merr.	Menispermaceae	Lagtang or Abutra	USTH 015600	922	10	2.75	3.23	2.14	Jaundice; tumor, myoma; diabetes, tonic; respiratory disease complex; diarrhea, dysentery, dyspepsia, ulcer, appetite enhancer; dysmenorrhea, delayed menstruation; abortifacient; fever	Rt, St	I	Drink decoction	3–5 glasses	Thrice a day or as needed	Can cause abortion in pregnant women
										Scabies; boils, skin rashes and itchiness; cuts and wounds	Rt, St	E	Apply coconut oil-infused stem	Completely on affected part	Once or twice a day or as needed	None
83	*Tinospora crispa* (L.) Hook. f. & Thomson	Menispermaceae	Panyawan	USTH 015566	782	9	2.33	2.68	1.95	Malaria; tonic; diarrhea, stomach trouble, vomiting, ulcer, toothache; arthritis, rheumatism; dysmenorrhea; abortifacient; abdominal pain, backache, body ache, fever	St	I	Drink local alcohol-tinctured or decocted stem	1–3 glasses	Once or twice a day or as needed	Can cause abortion in pregnant women
										Scabies; sore eyes; cuts and wounds	Sp, St	E	Drop stem sap	Completely on affected part	As needed	None
										Arthritis, rheumatism; abortifacient; abdominal pain, body ache; gas pain and flatulence	St	E	Apply coconut oil-infused stem or stem mixed with gasoline	Completely on affected part	As needed	Can cause abortion in pregnant women
84	*Ficus botryocarpa* Miq*.*	Moraceae	Kabiya	USTH 015672	53	1	0.16	0.96	0.00	Headache, fever	Rt	I	Drink decoction	1 arm-sized root	Twice a day or as needed	None
85	*Ficus cassidyana* Elmer	Moraceae	Tobog tapol	USTH 015551	492	8	1.47	3.00	1.89	Colds; diabetes; hypertension; asthma, cough, respiratory disease complex; diarrhea, stomachache; urinary tract infection; postpartum recovery, maternal care, milk production enhancer; weakness and fatigue, relapse	Bk, Rt	I	Drink decoction	1–3 glasses	Thrice a day or as needed	None
										Diabetes; hypertension	Fr	I	Eat fresh fruit directly	1–3 fruits	Once a day or as needed	None
										Body ache, headache, fever	Lf	E	Apply leaves as poultice	3–5 leaves	As needed	None
86	*Ficus concinna* (Miq.) Miq.	Moraceae	Balete	USTH 015552	608	4	1.81	2.66	1.37	Prostate cancer, cyst, tumor; arthritis, rheumatism; kidney problem, prostate problem	Bk, Lf, Rt	I	Drink decoction	5–7 glasses	Once a day in thrice a week for 2 months	In excess can cause anemia, dizziness, and weakening
										Cuts and wounds	Bk, Lf, Rt	E	Apply decoction as wash	1–3 glasses	As needed	None
										Fracture and dislocation, sprain	Bk, Rt	E	Apply fresh and heated bark and root as poultice	1–3 palm-sized barks or 1 arm-sized root	Once a day or as needed	Bark can cause skin allergy or burn
87	*Ficus fistulosa* Reinw. ex Blume	Moraceae	Tobog puti	USTH 015561	480	8	1.43	1.62	1.89	Colds; diabetes; hypertension; asthma, cough, respiratory disease complex; diarrhea, stomachache; urinary tract infection; maternal care, postpartum recovery, milk production enhancer; weakness and fatigue, relapse	Bk, Rt	I	Drink decoction	1–3 glasses	Thrice a day or as needed	None
										Diabetes; hypertension	Fr	I	Eat fresh fruit directly	1–3 fruits	Once a day or as needed	None
										Body ache, headache, fever	Lf	E	Apply leaves as poultice	3–5 leaves	As needed	None
88	*Ficus pseudopalma* Blanco	Moraceae	Lobi-lobi	USTH 015636	331	7	0.99	2.38	1.80	Diabetes; hypertension, atherosclerosis, hemorrhage; diarrhea, stomach trouble, dyspepsia; kidney stones; muscle pain; postpartum care and recovery; cramp and spasm	Lf, Rt	I	Drink decoction	3–5 glasses	Twice a day or as needed	None
89	*Ficus septica* Burm.f.	Moraceae	Lagnob	USTH 015623	576	7	1.72	2.13	1.77	Stomach trouble; asthma, sinusitis; muscle pain; backache, body ache, headache, fever, weakness, and fatigue	Lf, Rt	I	Drink decoction	3–5 glasses	Once a day for 3 days only	In excess can cause intestinal weakening
										Warts; cataract, eye problem	Lf, Sp	E	Apply drops of leaf sap	3–5 leaves	Twice a day or as needed	None
										Herpes simplex; boils	Rt, Sp, St	E	Apply stem and root sap	Completely on affected part	Thrice a day or as needed	None
90	*Ficus* sp.	Moraceae	Tuwa-tuwa	USTH 015642	71	1	0.21	0.98	0.00	Pregnancy, impotence and sterility, postpartum care and recovery	Lf	I	Drink decoction	3–5 glasses	Once a day for 3–5 days or as needed	None
91	*Morus alba* L.	Moraceae	Tahibo	USTH 015549	277	4	0.83	2.69	1.21	Colds; asthma, pneumonia, lung nodule, cough; muscle pain; cramp and spasm, relapse	Lf	I	Drink decoction	3–5 glasses	Twice to thrice a day for 3–5 days	In excess can cause acid reflux and hypocupremia
92	*Muntingia calabura* L.	Muntingiaceae	Mansanitas	USTH 015629	169	4	0.50	1.58	1.21	Colds; diarrhea, stomachache, vomiting, ulcer; prostate problem; abdominal pain, headache	Lf	I	Drink decoction	3–5 glasses	Twice a day or as needed	None
93	*Myristica agusanensis* Elmer	Myristicaceae	Duguang kahoy	USTH 015611	194	2	0.58	1.96	0.69	Measles; respiratory disease complex	Bk	I	Drink decoction	3–5 glasses	Once a day or as needed	None
94	*Psidium guajava* L.	Myrtaceae	Bayabas	USTH 015663	275	5	0.82	2.15	1.43	Diarrhea, ulcer	Bk, Lf, Rt	I	Drink decoction	3–5 glasses	Once to thrice a day or as needed	None
										Constipation	Fr	I	Eat fresh fruit directly	1–3 fruits	Once to thrice a day or as needed	None
										Scabies; toothache; skin eruptions; cuts and wounds	Lf	E	Apply chewed or pounded leaves	3–5 leaves	Once to thrice a day or as needed	None
										Dandruff; cuts and wounds; circumcision antiseptic	Lf	E	Apply decocted leaves as wash	3–5 leaves	Once to thrice a day or as needed	None
95	*Pandanus amaryllifolius* Roxb.	Pandanaceae	Pandantsina	USTH 015555	197	2	0.59	1.98	0.60	Heart enlargement, high cholesterol; urination difficulty, kidney stone, kidney problem, urinary bladder swelling, prostate problem	Lf	I	Drink decoction	3–5 glasses	Once to thrice a day or as needed	None
96	*Phyllanthus amarus* Schumach. & Thonn.	Phyllanthaceae	Talikod or Likod-likod	USTH 015590	459	9	1.37	2.35	2.01	Jaundice, colds; tonic; coughs; stomach problem; kidney stone, kidney problem, urinary bladder swelling; new-born baby care; fever	Wh	I/E	Drink decoction or apply decocted leaves as wash	3–5 glasses decoction or 1/2 cup leaf sap (adult); 1/2 cup decoction or 1 teaspoonful leaf sap (baby)	Once or twice a day or as needed	None
										Scabies, jaundice, ringworm; skin rashness and itchiness, dermatitis, eczema; cuts and wounds	Fr, Rt	E	Apply decoction as wash	3–5 glasses	Thrice a day or as needed	None
97	*Piper aduncum* L.	Piperaceae	Lunas buyo	USTH 015568	193	2	0.58	1.14	0.56	Skin rashes and itchiness; cuts and wounds, animal and insect bites	Lf, St	E	Apply decoction	Completely on affected part	Once or twice a day or as needed	In excess can cause intestinal weakening
98	*Piper decumanum* L.	Piperaceae	Lunas bagon tapol	USTH 015544	1018	9	3.04	3.45	2.06	Typhoid fever; cancer, cyst, tumor; pulmonary tuberculosis; diarrhea, stomach trouble, ulcer; gas pain and flatulence; poisoning	St	I	Drink local alcohol-tinctured stem	1/2 to 1 glass	Once a day in thrice a week or as needed	In excess can cause intestinal weakening
										Tonsillitis; toothache, gum swelling, canker sore	St	I	Gargle local alcohol-tinctured stem	1/2 to 1 glass	Once or twice a day or as needed	None
										Scabies, warts, impetigo; boils, skin eruptions, skin rashes and itchiness, pimple, acne; arthritis, rheumatism, swellings, muscle pain; backache, body ache, gas pain and flatulence; allergy, burns, cuts and wounds, sprain, snake, dog and insect bites, contacts with plants and animal parts; anesthetic	St	E	Apply coconut or Efficascent oil-infused stem	Completely on affected part	Once or twice a day or as needed	None
99	*Piper nigrum* L.	Piperaceae	Lunas bagon puti	USTH 015560	824	9	2.46	2.41	1.20	Cancer, cyst, tumor; tonsillitis; diarrhea, stomach trouble, ulcer, toothache, mouth sore, gum swelling; gas pain and flatulence; poisoning	St	I	Drink or gargle local alcohol-tinctured stem or drink decocted stem	1/2 to 1 glass of local alcohol tincture or 3–5 glasses of decoction	Once a day in thrice a week or as needed	In excess can cause intestinal weakening
										Scabies, warts, impetigo; breast cancer; boils, skin eruptions, skin rashes and itchiness, pimple, acne; arthritis, rheumatism, swellings, muscle pain; backache, body ache, gas pain, and flatulence; skin allergy, burns, cuts and wounds, animal and insect bites, contacts with plants and animals parts; anesthetic	St	E	Apply coconut oil-infused or decocted stem	Completely on affected part	Once or twice a day or as needed	None
100	*Piper* sp.	Piperaceae	Buyo Pilipog	USTH 015592	296	3	0.88	1.33	1.04	Asthma, cough; rheumatism; fracture and dislocation	Lf	I	Drink decoction	3–5 glasses	Once a day or as needed	None
101	*Eleusine indica* (L.) Gaertn.	Poaceae	Bilabila	USTH 015569	481	11	1.44	3.04	2.34	Measles; diabetes; internal bleeding; cough; diarrhea; arthritis; kidney problem; postpartum care and recovery; fever, cramp, and spasm; fracture and dislocation	Wh	I	Drink decoction	3–5 glasses	Thrice a day or as needed	None
										Ringworm; hair loss; cuts and wounds	Wh	E	Apply decoction	1 glass	Once to thrice a day or as needed	None
102	*Imperata cylindrica* (L.) P.Beauv.	Poaceae	Kogon	USTH 015605	107	4	0.32	1.90	1.35	Urination difficulty	Sh	I	Drink decoction	3–5 glasses	Once to thrice a day or as needed	None
										Chicken pox, measles; diarrhea, toothache; fever, baby teething	Rt	I	Drink decoction	3–5 glasses	Once to thrice a day or as needed	None
103	*Paspalum conjugatum* P.J.Bergius	Poeaceae	Miligoy	USTH 015627	124	3	0.37	2.30	1.04	Diarrhea, dysentery	Rt	I	Drink decoction	1 handful of roots	Twice a day or as needed	None
										Hair loss; cuts and wounds	Rt	E	Apply decoction as wash	1 handful of roots	Once a day or as needed	None
104	*Rosa* sp.	Rosaceae	Rose	USTH 015628	83	3	0.25	1.33	1.04	Colds; nasal congestion, sinusitis	Fl	I	Drink or sniff hot water-infused flowers	1 glass	Thrice a day or as needed	None
										Cuts and wounds	Fl	E	Apply hot water-infused flowers as wash	1 glass	As needed	None
105	*Mussaenda philippica* A.Rich.	Rubiaceae	Buyon	USTH 015556	123	5	0.37	1.36	1.56	Jaundice, colds; dysentery, stomachache; fever; snake bite	Bk, Lf	I	Drink decoction	3–5 glasses	Once to thrice a day or as needed	None
										Asthma, cough	Fl	I	Eat fresh flower directly	1–3 flowers	Once or twice a day or as needed	None
106	*Uncaria lanosa* Wall.	Rubiaceae	Kawilan	USTH 015557	94	1	0.28	0.93	0.00	Stomach trouble	Sp, St	I	Drink stem sap	1/2 cup	Once or twice a day or as needed	None
										Diarrhea	St	I	Drink local alcohol-tinctured bark	1/2 to 1 glass	As needed	None
107	*Melicope latifolia* (DC.) T.G.Hartley	Rutaceae	Bagaynga	USTH 015540	43	1	0.13	0.92	0.00	Cough	Bk	I	Drink decoction of scraped bark	1–3 palm-sized barks	Once or twice a day or as needed	None
108	*Melicope triphylla* (Lam.) Merr.	Rutaceae	Dahile	USTH 015660	86	2	0.26	1.31	0.64	Pulmonary tuberculosis, cough	Lf, Sp	I	Drink decoction or leaf sap	3–5 glasses or 3–5 leaves of sap	Once to thrice a day or as needed	None
										Snake bite	Lf, Sp	E	Apply leaf sap	Completely on affected part	As needed	None
109	*Micromelum minutum* (G.Forst.) Wight & Arn.	Rutaceae	Lunas kahoy	USTH 015538	955	9	2.85	3.28	2.03	Cancer, cyst; diarrhea, stomach trouble, ulcer; poisoning	Lf, St	I	Drink local alcohol-tinctured or decocted stem	1/2 to 1 glass	Once or twice a day or as needed	In excess can cause intestinal weakening
										Tonsillitis; toothache, gum swelling, canker sore	Rt, St	E	Gargle local alcohol-tinctured stem	1/2 to 1 glass	Once or twice a day or as needed	None
										Scabies, warts, impetigo; boils, skin eruptions, skin rashes and itchiness, pimple, acne; joint pain, rheumatism, swellings, muscle pain; backache, body ache, gas pain, and flatulence; allergy, burns, cuts, and wounds; snake, dog, and insect bites; contacts with plants and animal parts; anesthetic	Rt, St	E	Apply coconut or Efficascent oil-infused stem	Completely on affected part	Once or twice a day or as needed	None
110	*Capsicum annuum* L.	Solanaceae	Sili na bisaya	USTH 015626	151	6	0.45	2.48	1.61	Diabetes; hypertension	Fr	I	Eat fresh fruit directly or add as spice in cooking	7 fruits	Once or twice a day or as needed	None
										White spot, athlete's foot; appetite enhancer; boils, skin rashes and itchiness, psoriasis, dandruff; insect bites	Lf	E	Rub crushed leaves or leaf sap	3–5 leaves	Once or twice a day or as needed	None
111	*Grewia laevigata* Vahl	Sparmanniaceae	Talimughat lingin	USTH 015547	474	5	1.41	1.75	1.42	Diabetes; hypertension, heart enlargement; rheumatism; labor and delivery enhancer, postpartum care and recovery; backache, body ache, fever, weakness, and fatigue, relapse	Bk, Lf, Rt	I	Drink decoction	3–5 glasses	Once to thrice a day up to 3 days or as needed	None
										Muscle pain; labor and delivery enhancer, postpartum care and recovery; backache, body ache, fever, cramp, and spasm; relapse	Bk, Rt	E	Apply coconut or Efficascent oil-infused bark and root	Completely on affected part	Once a day or as needed	None
112	*Dendrocnide luzonensis* (Wedd.) Chew	Urticaceae	Alingatong	USTH 015598	128	2	0.38	2.33	0.56	Diabetes; joint pain, swollen muscles, and swellings, muscle pain	Rt	I	Drink decoction	3–5 glasses	Once to thrice a day or as needed	None
113	*Leucosyke capitellata* Wedd.	Urticaceae	Anagasi	USTH 015542	28	1	0.08	0.96	0.00	Stomach trouble and vomiting	Lf	I	Drink decoction	3–5 glasses	Once or twice a day or as needed	None
114	*Oreocnide rubescens* (Blume) Miq.	Urticaceae	Kubi or Salin-ubod	USTH 015676	151	3	0.45	1.89	1.04	Cough; diarrhea, stomach trouble; fever	Lf	I	Drink decoction	3–5 glasses	Twice a day or as needed	None
115	*Pipturus arborescens* (Link) C.B.Rob.	Urticaceae	Handamay	USTH 015673	540	6	1.61	2.25	1.58	Depression, anxiety, nervousness; stomach acidity; backache, body ache, headache, fever, weakness, and fatigue	Lf	I	Eat fresh leaves or drink water solution of leaves	3–5 glasses	Thrice a day for a month or as needed	None
										Herpes simplex, scabies; boils, dermatitis	Lf	E	Rub crushed leaves	3–5 leaves	Thrice a day or as needed	None
										Skin rashes and infection; cuts and wounds	Bk	E	Apply pulp made from scraped bark as poultice	1–3 palm-sized barks	Twice a day or as needed	None
116	*Poikilospermum acuminatum* (Trecul.) Merr.	Urticaceae	Hanupi	USTH 015655	243	6	0.73	2.06	1.67	Sore eyes; ulcer; postpartum care and recovery; fever	Sp, St	I	Drink stem sap or decoction stem	1 arm-sized stem	Once or twice a day or as needed	None
										Skin rashes and itchiness; cuts and wounds; animal and insect bites	Rt, Sp	E	Apply decoction as wash	1 arm-sized root	Once or twice a day or as needed	None
117	*Stachytarpheta jamaicensis* (L.) Vahl	Verbenaceae	Elepanteng lingganag	USTH 015594	396	5	1.18	2.04	1.56	Ascariasis; abortifacient; fever	Rt	I	Drink decoction	1 arm-sized root	Once to thrice a day or as needed	Can cause abortion in pregnant women
										Boils; bruises, sprain	Lf	E	Apply crushed leaves as poultice	3–5 glasses	As needed	None
118	*Dianella ensifolia* (L.) DC.	Xanthorrhoeaceae	Ikug-ikug	USTH 015656	164	3	0.35	1.39	0.95	Maternal care; postpartum care and recovery, milk production enhancer	Lf	I	Drink decoction	3–5 glasses	Once a day or as needed	None
										Cuts and wounds	Lf	E	Apply leaves as poultice	3–5 leaves	As needed	None
										Herpes simplex	Lf, Rt	E	Apply coconut oil-infused ashes of leaf and roots	Completely on affected part	Thrice a day or as needed	None
119	*Curcuma longa* L.	Zingerberaceae	Duwaw yellow	USTH 015674	248	6	0.74	1.68	1.70	Diabetes; cough; arthritis, rheumatism; delayed menstruation; fever, gas pain and flatulence	Rz	I	Drink grinded and brewed rhizome	1–3 cups	Once or twice a day or as needed	None
										Burns, cuts and wounds, insect bites	Rz	E	Apply extracted juice from crushed rhizome	Completely on affected part	As needed	None
120	*Curcuma zedoaria* (Christm.) Rosc.	Zingerberaceae	Duwaw violet	USTH 015645	83	2	0.24	1.88	0.69	Cough; fever	Rz	I	Drink grinded and brewed rhizome	1–3 cups	Once or twice a day or as needed	None
121	*Alpinia haenkei* C.Presl	Zingiberaceae	Yanguas	USTH 015641	86	3	0.26	1.26	1.04	Cough; stomachache; urination difficulty, urinary tract infection	Rt	I	Drink decoction	3–5 glasses	Once to thrice a day or as needed	None
122	*Kaempferia galanga* L.	Zingiberaceae	Kisol	USTH 015579	200	7	0.60	1.44	1.89	Colds; tonic; cough, sore throat; dyspepsia, toothache; postpartum care and recovery; headache, fever	Rz	I	Drink decoction	3–5 glasses	Twice a day or as needed	None
										Toothache; headache, fever	Rz	I/E	Apply grinded rhizome as poultice	1–3 rhizomes	Thrice a day or as needed	None
										Tonic; postpartum care and recovery; headache, fever	Sh	E	Place washed clean shoot around the neck	1–3 shoots	Once a day or as needed	None
										Cough; rheumatism, swollen muscle	Lf	E	Rub heated and crashed leaves	1–3 leaves	Thrice a day or as needed	None

*UR* use-report, *UC* use category, *UV* use value, *CIV* cultural importance value, *UD* use diversity

^a^*Bk*, barks; *Br*, branch; *Fl*, flowers; *Fr*, fruits; *Lf*, leaves; *Rt*, roots; *Rz*, rhizomes; *Sd*, seeds; *Sh*, shoots; *St*, stems; *Wh*, whole plant

^b^*I*, internal; *E*, external

### Integrative molecular approach

Due to inconclusive morphological identification, unfamiliarity, and confusing species identity because of local name similarity, a total of 24 medicinal plant species were confirmed by DNA sequencing and by comparing the sequences with those present in the GenBank. This method supported ethnopharmacological data to be deposited in a repository, which is essential and helpful for future researchers and investigators for use by data mining approaches [73]. The molecular data can also be useful to the growing barcoding studies of medicinal plants. Putative identification based on literature, comparative morphology, and molecular sequences using the BLAST search query were tabulated (Table 5). The integrative approach combined with a priori data from putative identifications based on the interview data on local or vernacular names, local plant name dictionary, and assessment of available morphological characteristics along with a posteriori data from multiple universal markers, occurrence, and distribution of putative species in the Philippines. This paper applied a more detailed taxonomic identification since all reported medicinal plant taxa were identified (nearly all to species level), as shown in Table 4. While all generic and familial affinities of medicinal plants were confirmed, four medicinal plants were not identified up to species level due to lack of morphological characteristics, concerning especially the reproductive parts of *Piper* and *Ficus* species, several cultivars and hybrids of *Rosa* species, and several species and varieties of *Bauhinia* species. Nevertheless, all generic and familial affinities of the medicinal plants documented here were verified combining similarity matching and a priori and a posteriori data as recommended by [42] to reduce ambiguity and to make it possible assigning a single species identification of their unidentifiable specimens. All determined plant samples with confusing identity having local name similarity and local species pairing, including plant samples with inconclusive morphological identification due to lack of reproductive parts upon collection, were accurately verified using an integrative molecular approach (Table 5).

**Table 5:** Integrative molecular identification coalescing a priori and a posteriori data

Taxon no.	Local name	Putative identification based on the *Dictionary of Philippine Plant Names* [51]	Species determination using present morphology	Molecular confirmation by BLAST-based sequence matching using multiple molecular markers																Integrative molecular identification approach
				ITS (nrDNA)				*mat*K (cpDNA)				*psb*A-*trn*H (cpDNA)				*trn*L-F (cpDNA)				
				Simple BLAST			Optimized BLAST [max score× (query cover/percent identity)]	Simple BLAST			Optimized BLAST [max score × (query cover/percent identity)]	Simple BLAST			Optimized BLAST [max score × (query cover/percent identity)]	Simple BLAST			Optimized BLAST [max score × (query cover/percent identity)]	
				Top 5 max score	Highest max score	Highest percent identity		Top 5 max core	Highest max score	Highest percent identity		Top 5 max score	Highest max score	Highest percent identity		Top 5 max score	Highest max score	Highest mercent identity		
1	Abgaw	*Premna odorata*	*Premna* sp.	*Premna* spp.	*Premna serratifolia*	*Premna serratifolia*	*Premna serratifolia*	*Premna* spp.	*Premna odorata*	*Premna serratifolia*	*Premna odorata*	*Premna* spp.	*Premna serratifolia*	*Premna odorata*	*Premna serratifolia*	*Premna* spp.	*Premna odorata*	*Premna odorata*	*Premna odorata*	*Premna odorata* Blanco
2	Alibangbang (puti)	*Bauhinia monandra. Bauhinia purpurea*	*Phanera* sp.	*Bauhinia* spp.	*Bauhinia nervosa*	*Bauhinia semibifida*	*Bauhinia touranensis*	NONE				Fabaceae	*Lasiobema championii*	*Lasiobema championii*	*Bauhinia nervosa*	Fabaceae	*Bauhinia yunnanensis*	*Bauhinia yunnanensis*	*Bauhinia yunnanensis*	*Phanera semibifida* (Roxb.) Benth.
3	Alibangbang (tapol)	*Bauhinia monandra. Bauhinia purpurea*	*Phanera* sp.	*Bauhinia* spp.	*Bauhinia nervosa*	*Bauhinia semibifida*	*Bauhinia nervosa*	NONE				Fabaceae	*Lasiobema championii*	*Lasiobema championii*	*Barklya syringifolia*	Fabaceae	*Bauhinia yunnanensis*	*Bauhinia yunnanensis*	*Bauhinia yunnanensis*	*Phanera semibifida* (Roxb.) Benth.
4	Awoy	None	*Callicarpa* cf. *pedunculata*	*Callicarpa* spp.	*Callicarpa formosana*	*Callicarpa formosana*	*Callicarpa rubella*	*Callicarpa* spp.	*Callicarpa bodinieri*	*Callicarpa giraldii*	*Callicarpa bodinieri*	*Callicarpa* sp.	*Callicarpa bodinieri*	*Callicarpa dichotoma*	*Callicarpa bodinieri*	Lamiaceae, Martyniaceae	*Callicarpa giraldii*	*Callicarpa giraldii*	*Craniolaria integrifolia*	*Callicarpa pedunculata* R.Br.
5	Balete	*Ficus* sp.	*Ficus* sp.	*Ficus* spp.	*Ficus concinna*	*Ficus glabella*	*Ficus cordata subsp. salicifolia*	*Ficus* spp.	*Ficus carica*	*Ficus carica*	*Ficus carica*	*Ficus* spp.	*Ficus pachyclada*	*Ficus simplicissima*	*Ficus carica*	*Ficus* spp.	*Ficus carica*	*Ficus carica*	*Ficus carica*	*Ficus concinna* (Miq.) Miq.
6	Banag	*Dioscorea* sp.	*Stenomeris* sp.	NONE				Dioscoreaceae	*Stenomeris borneensis*	*Stenomeris borneensis*	*Stenomeris borneensis*	Dioscoreaceae, Arecaceae	*Dioscorea brachybotrya*	*Corypha lecomtei*	*Dioscorea brachybotrya*	Dioscoreaceae and Blandfordiaceae	*Stenomeris borneensis*	*Stenomeris borneensis*	*Stenomeris borneensis*	*Stenomeris borneensis* Oliv*.*
7	Banitlong	*Melochia umbellata*	*Melochia* sp.	Malvaceae	*Waltheria indica*	*Waltheria* sp.	*Waltheria indica*	Convolvulaceae and Malvaceae	*Ipomoea setifera*	*Ipomoea setifera and Waltheria indica*	*Ipomoea setifera*	Convolvulaceae and Malvaceae	*Waltheria indica*	*Ipomoea setifera*	*Waltheria indica*	Convolvulaceae and Malvaceae	*Ipomoea setifera*	*Ipomoea setifera*	*Ipomoea setifera*	*Melochia umbellata* (Houtt.) Stapf
8	Banti (puti)	*Homalanthus populneus*	*Omalanthus* sp.	Euphorbiaceae	*Homalanthus nutans*	*Homalanthus nutans*	*Triadica sebifera*	Euphorbiaceae	*Homalanthus populneus*	*Homalanthus nutans*	*Homalanthus populneus*	Ebenaceae, Euphorbiaceae	*Diospyros geminata*	*Triadica sebifera*	*Diospyros geminata*	Euphorbiaceae	*Homalanthus populneus*	*Homalanthus populneus*	*Homalanthus populneus*	*Omalanthus macradenius* Pax & Hoffm.
9	Banti (tapol)	*Homalanthus populneus*	*Omalanthus* sp.	Euphorbiaceae	*Homalanthus nutans*	*Homalanthus nutans*	*Triadica sebifera*	Euphorbiaceae	*Homalanthus populneus*	*Homalanthus nutans*	*Homalanthus populneus*	Ebenaceae, Lauraceae	*Diospyros geminata*	*Cinnamomum* sp.	*Diospyros geminata*	Euphorbiaceae	*Homalanthus populneus*	*Homalanthus populneus*	*Homalanthus populneus*	*Omalanthus macradenius* Pax & Hoffm.
10	Gapas-gapas (bae)	*Camptostemon philippinense*	*Erechtites* sp.	Compositae	*Erechtites valerianifolia*	*Erechtites valerianifolia*	*Erechtites valerianifolia*	Compositae	*Jacobaea erucifolia*	*Jacobaea erucifolia*	*Jacobaea erucifolia*	Compositae	*Erechtites valerianifolius*	*Erechtites hieraciifolius*	*Erechtites valerianifolius*	Compositae	*Erechtites valerianifolius*	*Erechtites valerianifolius*	*Erechtites valerianifolius*	*Erechtites valerianifolius* (Link ex Spreng.) DC.
11	Kaningag	*Cinnamomum celebicum*	*Cinnamomum* cf. mercadoi	*Cinnamomum* spp.	*Cinnamomum paiei*	*Cinnamomum paiei*	*Cinnamomum paiei*	Lauraceae	*Cinnamomum reticulatum*	*All are equal*	*Cinnamomum reticulatum*	Lauraceae	*Cinnamomum verum*	*Cinnamomum verum*	*Cinnamomum verum*	*Cinnamomum* spp.	*Cinnamomum insularimontanum*	*Cinnamomum insularimontanum*	*Cinnamomum insularimontanum*	*Cinnamomum mercadoi* S.Vidal
12	Kawilan	*Uncaria laevifolia*	*Uncaria* cf. *lanosa*	*Uncaria macrophylla*	*Uncaria macrophylla*	*Uncaria macrophylla*	*Uncaria macrophylla*	Rubiaceae	*Uncaria lancifolia*	*Uncaria cf. scandens*	*Neolamarckia cadamba*	Rubiaceae	*Uncaria laevigata*	*Uncaria laevigata*	*Uncaria laevigata*	Rubiaceae	*Uncaria lanosa*	*Uncaria lanosa*	*Uncaria rhynchophylla*	*Uncaria lanosa* Wall.
13	Lunas-bagon (puti)	None	*Piper* sp.	*Piper subcaniramum*	*Piper subcaniramum*	*Piper subcaniramum*	*Piper cathayanum*	*Piper* spp.	*Piper chinense*	*Piper chinense*	*Piper chinense*	*Piper* spp.	*Piper nigrum*	*Piper kadsura*	*Piper nigrum*	*Piper* spp.	*Piper nigrum*	*Piper mullesua*	*Piper nigrum*	*Piper nigrum* L.
14	Mayana Kanapkap	*Coleus blumei*	*Plectranthus* sp.	Lamiaceae	*Isodon japonicus*	*Plectranthus barbatus*	*Isodon japonicus*	NONE				Lamiaceae	*Plectranthus scutellarioides*	*Plectranthus scutellarioides*	*Ocimum gratissimum*	*Plectranthus* spp.	*Solenostemon scutellarioides,* syn. *of Plectranthus scutellarioides*	*Solenostemon scutellarioides*	*Plectranthus fredricii*	*Coleus scutellarioides* (L.) Benth.
15	Mayana Pula	*Coleus blumei*	*Plectranthus* sp.	*Isodon* spp.	*Isodon japonicus*	*Isodon japonicus*	*Isodon japonicus*	NONE				Lamiaceae	*Plectranthus scutellarioides*	*Plectranthus scutellarioides*	*Ocimum gratissimum*	*Plectranthus* spp.	*Solenostemon scutellarioides,* syn. of *Plectranthus scutellarioides*	*Solenostemon scutellarioides,* syn. of *Plectranthus scutellarioides*	*Solenostemon scutellarioides,* syn. of *Plectranthus scutellarioides*	*Coleus scutellarioides* (L.) Benth.
16	Salimbagat	*Capparis micracantha*	*Thottea* cf. *affinis*	NONE				*Thottea* spp.	*Thottea penitilobata, Thottea borneensis and Thottea dependens*	*Thottea penitilobata, Thottea borneensis and Thottea dependens*	*Thottea penitilobata, Thottea borneensis and Thottea dependens*	*Thottea* spp.	*Thottea hainanensis*	*Thottea hainanensis*	*Thottea hainanensis*	Aristolochiaceae	*Thottea siliquosa*	*Thottea siliquosa*	*Thottea siliquosa*	*Thottea affinis* (Planch. ex Rolfe) ined.
17	Talimughat 1 (lingin)	*Oxymitra paucinervia*	*Grewia* cf. *laevigata*	*Grewia* spp.	*Grewia trichocarpa*	*Grewia biloba*	*Grewia trichocarpa*	*Grewia* spp.	*Grewia biloba*	*Grewia lasiocarpa*	*Grewia biloba*	Malvaceae	*Pterygota alata*	*Pterygota alata*	*Pterygota alata*	Malvaceae	*Microcos paniculata*, syn. of *Grewia nervosa*	*Microcos paniculata,* syn. of *Grewia nervosa*	*Microcos paniculata*, syn. of *Grewia nervosa*	*Grewia laevigata* Vahl
18	Talimughat 2 (taas)	*Oxymitra paucinervia*	*Friesodielsia* cf. *lanceolata*	NONE				Annonaceae	*Friesodielsia* spp.	*Friesodielsia desmoides*	*Uvaria macrophylla*	Annonaceae	*Friesodielsia* sp.	All are equal	*Friesodielsia* sp.	Annonaceae	*Monanthotaxis aquila*	*Monanthotaxis aquila, Monanthotaxis pellegrinii,* and *Friesodielsia* sp.	*Monanthotaxis aquila*	*Friesodielsia lanceolata* (Merr.) Steen.
19	Talimughat 3 (pikas)	*Oxymitra paucinervia*	*Bauhinia* sp.	*Bauhinia* spp.	*Bauhinia touranensis*	*Bauhinia kockiana*	*Bauhinia touranensis*	NONE				Fabaceae	*Lasiobema championii*	*Lasiobema championii*	*Lasiobema championii*	*Bauhinia* spp.	*Phanera bidentata*	*Phanera bidentata*	*Phanera bidentata*	*Bauhinia* sp.
20	Tobog (puti)	*Ficus botryocarpa*	*Ficus* cf. *fistulosa*	*Ficus* spp.	*Ficus fistulosa*	*Ficus fistulosa*	*Ficus fistulosa*	*Ficus* spp.	*Ficus carica*	*Ficus carica*	*Ficus carica*	*Ficus* spp.	*Ficus hirta*	*Ficus hirta* and *Ficus trigonata*	*Ficus religiosa*	*Ficus* spp.	*Ficus carica*	*Ficus carica*	*Ficus carica*	*Ficus fistulosa* Reinw. ex Blume
21	Tobog (tapol)	*Ficus botryocarpa*	*Ficus* cf. *cassidyana*.	*Ficus* spp.	*Ficus lepicarpa*	*Ficus lepicarpa*	*Ficus lepicarpa*	*Ficus* spp.	*Ficus carica*	*Ficus carica*	*Ficus carica*	*Ficus* spp.	*Ficus trigonata*	*Ficus trigonata*	*Ficus religiosa*	*Ficus* spp.	*Ficus carica*	*Ficus carica*	*Ficus carica*	*Ficus cassidyana* Elmer
22	Tuba-tuba (puti)	*Jatropha curcas, Jatropha gossypiifolia*	*Jatropha* cf. *curcas*	*Jatropha curcas*	*Jatropha curcas*	*Jatropha curcas*	*Jatropha curcas*	NONE				*Jatropha* spp.	*Jatropha curcas*	*Jatropha curcas*	*Jatropha curcas*	*Jatropha curcas*	*Jatropha curcas*	*Jatropha curcas*	*Jatropha curcas*	*Jatropha curcas* L.
23	Tuba-tuba (tapol)	*Jatropha curcas, Jatropha gossypiifolia*	*Jatropha* cf. *gossypiifolia*	*Jatropha* spp.	*Jatropha gossypiifolia*	*Jatropha gossypiifolia*	*Jatropha gossypiifolia*	*Jatropha* spp.	*Jatropha gossypiifolia*	*Jatropha podagrica*	*Jatropha gossypiifolia*	*Jatropha* spp.	*Jatropha gossypiifolia*	*Jatropha gossypiifolia*	*Jatropha gossypiifolia*	*Jatropha* spp.	*Jatropha gossypiifolia*	*Jatropha gossypiifolia*	*Jatropha gossypiifolia*	*Jatropha gossypifolia* L.
24	Tuwa-tuwa	None	*Ficus* cf. *ingens*	*Ficus* spp.	*Ficus glabella*	*Ficus glabella*	*Ficus ingens*	NONE				*Ficus* spp.	*Ficus carica*	*Ficus simplicissima, Ficus hirta*	*Ficus carica*	Moraceae	*Ficus pumila*	*Ficus pumila*	*Ficus pumila*	*Ficus* sp.

*NONE* unsuccessfully amplified and/or sequenced

### Plant local name similarity

Most notable medicinal plants of *Agusan Manobo* have confusing species identity bearing similar local names, gender identity, and local species pairing. It is popular to use medicinal plants known as “Lunas” (meaning “cure”) with several plants associated under its name. For instance, the top three medicinal plants in terms of use value and cultural importance value have local name similarity, namely Lunas tag-uli (*Anodendron borneense* (King & Gamble) D.J.Middleton), Lunas bagon tapol (*Piper decumanum* L.), and Lunas kahoy (*Micromelum minutum* (G.Forst.) Wight & Arn.), respectively. These three medicinal plants with the initial word named “Lunas” had almost similar use-reports in nine use categories with high use diversity (UD > 2.0). Other “Lunas”-named specimens such as Lunas bagon puti (*Piper nigrum* L.), Lunas pilipo (*Acmella grandiflora* (Turcz.) R.K.Jansen), Lunas buyo (*Piper aduncum* L.), and Lunas gabi (*Alocasia zebrina* Schott ex Van Houtte) also shared similarities from the top three mentioned samples in terms of ethnomedicinal properties as a treatment for cuts and wounds. Also, another three medicinal plants were locally classified with the initial word named “Talimughat” (meaning “recover”), namely “Talimughat lingin” (*Grewia laevigata* Vahl), “Talimughat taas” (*Friesodielsia lanceolata* (Merr.) Steen.), and “Talimughat pikas” (*Bauhinia* sp.). These three medicinal plants were noted with high fidelity for postpartum care and recovery. Plant samples with high fidelity for anemia also had similar local names which were found to be same species, namely “Mayana kanapkap” (*Coleus scutellarioides* (L.) Benth*.*) and “Mayana pula” (*Coleus scutellarioides* (L.) Benth*.*).

Some medicinal plants also have attached “genders” (male or female) in their local names, which specify the more effective plant “gender” for a specific medicinal use or purpose. Examples are “Kapayas laki” (*Carica papaya* L., male), “Dupang bae” (*Urena lobata* L., female), and “Gapas-gapas bae” (*Erechtites valerianifolius* (Link ex Spreng.) DC*.*, female) as effective treatments for dengue virus, postpartum care and recovery, and gas pain and flatulence, respectively. Besides, most species with high use values had local species pairing which were classified by the tribe according to distinct white and red coloration, namely “puti” and “tapol,” respectively, with the latter as more effective than the former in treatment for various health conditions. The following recognized local species pairs as white and red plant samples, respectively, are “Alibangbang puti” (*Phanera semibifida* (Roxb.) Benth.) and “Alibangbang tapol” (*Phanera semibifida* (Roxb.) Benth.); “Banti puti” (*Omalanthus macradenius* Pax & Hoffm.) and “Banti tapol” (*Omalanthus macradenius* Pax & Hoffm.); “Lunas-bagon puti” (*Piper nigrum*) and “Lunas-bagon tapol” (*Piper decumanum*); “Tobog puti” (*Ficus fistulosa* Reinw. ex Blume) and “Tobog tapol” (*Ficus cassidyana* Elmer); and “Tuba-tuba puti” (*Jatropha curcas* L.) and “Tuba-tuba tapol” (*Jatropha gossypifolia* L.). Local species pairing of “Alibangbang puti” and “Alibangbang tapol” was found to be similar species (*Phanera semibifida* (Roxb.) Benth.). Another species pair, “Banti puti” and “Banti tapol” was also found to be similar species (*Omalanthus macradenius* Pax & Hoffm.). However, molecular confirmation of all species pairs by the locals did not necessarily point to the same species but were mostly referring to another species. An example study resolving species identity of *Piper* species used by the *Agusan Manobo* being a sterile species and unidentifiable by present morphology having confusing local names with the initial word “Lunas” has been molecularly confirmed lately using integrative molecular approach [19]. Thus, it is always important in any ethnomedicinal, ethnobotanical, and ethnopharmacological studies to obtain the correct identification of medicinal plants by integrating molecular data like this for accuracy, consistency, and dependable species identity for future pharmacological evaluation and natural product investigations.

### Species molecular confirmation

Most of all extracted samples for molecular analysis were successfully amplified and sequenced (90%) using multiple universal markers (Table 5). Some medicinal plants could not be successfully amplified using the given primer due to low levels of DNA present in the samples [74] or plant secondary metabolites present as inhibitory factors [75]. Molecular data obtained were also subject to the availability of sequences of plant samples in the GenBank. The 24 species identified were tabulated in Table 6, showing six endemic species (27.3%) [56] and conservation status of all assessed species (37.5%) [76, 77] presented five least concern species (83.3%) and a vulnerable species, *Cinnamomum mercadoi* S.Vidal (16.7%). All edited sequences of each of the four DNA markers in *fasta* file format were attached as supplementary materials (see Additional files 2–5) for future reference.

**Table 6:** The 24 molecularly confirmed species with confusing species identity

Taxon no.	Local name	Family	Species	Endemicity [56]	Conservation status
1	Abgaw	Lamiaceae	*Premna odorata* Blanco		LC [76]
2	Alibangbang (puti)	Fabaceae	*Phanera semibifida* (Roxb.) Benth.		
3	Alibangbang (tapol)	Fabaceae	*Phanera semibifida* (Roxb.) Benth.		
4	Awoy	Lamiaceae	*Callicarpa pedunculata* R.Br.		LC [77]
5	Balete	Moraceae	*Ficus concinna* (Miq.) Miq.		LC [78]
6	Banag	Dioscoreaceae	*Stenomeris borneensis* Oliv.		
7	Banitlong	Byttneriaceae	*Melochia umbellata* (Houtt.) Stapf		
8	Banti (puti)	Euphorbiaceae	*Omalanthus macradenius* Pax & Hoffm.	EN	
9	Banti (tapol)	Euphorbiaceae	*Omalanthus macradenius* Pax & Hoffm.	EN	
10	Gapas-gapas (bae)	Asteraceae	*Erechtites valerianifolius* (Link ex Spreng.) DC.		
11	Kaningag	Lauraceae	*Cinnamomum mercadoi* S.Vidal	EN	VU [78]
12	Kawilan	Rubiaceae	*Uncaria lanosa* Wall.		
13	Lunas-bagon (puti)	Piperaceae	*Piper nigrum* L.		
14	Mayana Kanapkap	Lamiaceae	*Coleus scutellarioides* (L.) Benth.		
15	Mayana Pula	Lamiaceae	*Coleus scutellarioides* (L.) Benth.		
16	Salimbagat	Aristolochiaceae	*Thottea affinis* (Planch. ex Rolfe) ined.	EN	
17	Talimughat 1 (lingin)	Sparmanniaceae	*Grewia laevigata* Vahl		LC [78]
18	Talimughat 2 (taas)	Annonaceae	*Friesodielsia lanceolata* (Merr.) Steen.	EN	
19	Talimughat 3 (pikas)	Fabaceae	*Bauhinia*sp.		
20	Tobog (puti)	Moraceae	*Ficus fistulosa* Reinw. ex Blume		LC [78]
21	Tobog (tapol)	Moraceae	*Ficus cassidyana* Elmer	EN	
22	Tuba-tuba (puti)	Euphorbiaceae	*Jatropha curcas* L.		
23	Tuba-tuba (tapol)	Euphorbiaceae	*Jatropha gossypifolia* L.		
24	Tuwa-tuwa	Asteraceae	*Ficus* sp.		

Endemicity: *EN* endemic

Conservation status: *LC* least concern, *VU* vulnerable

The most certain identity confirmed by this molecular analysis is the familial and generic affinity wherein the specific epithet of each of the 24 medicinal plants presented had to be verified for its occurrence and distribution in the country. All species identified using simple and optimized BLAST-based sequence matching results were further reviewed on their present morphology using taxonomic keys and comparing images and specimens before consulting an expert. Some species names presented in BLAST search query have synonyms showing similar genus among species within 5 points deviation down of the max score. In contrast, others have several genera but under the same family. Two species with molecular data, namely *Bauhinia* sp. and *Ficus* sp., were only confirmed up to the genus level due to limited morphological material and because of a high number of varieties, species, and subspecies. A sterile *Piper* species was confirmed as *P. nigrum* based on its diagnostic characterization, which could be a new variety obtained only in the wild among the respondents and not the widely cultivated spice known as the world’s most consumed peppercorn.

Of all DNA markers used in this study, two markers, *psb*A-*trn*H and *trn*L-F (cpDNA) successfully amplified and sequenced all 24 uncertain species (100%). A total of 21 species (88%) were amplified and sequenced using the marker ITS (nrDNA), while the coding marker, *mat*K (cpDNA), recorded at least 17 amplified and sequenced species (71%). In this case, molecular data could increase its identification rate by using multiple universal markers. Several coding and non-coding regions were tested in plants, but a single locus has limited resolving capabilities for closely related species [79, 80]. While local names are essential in ethnopharmacological studies, complexities of these local names could lead to confusion and ambiguity, hence, a need for further molecular analysis [19]. A number of ethnobotanical studies consider vernacular names coupled with morphological and molecular confirmation as part of the identification diagnostics [19, 42, 81–83].

### Collection sites

The majority (57%) of the medicinal plants were collected in the wild, while some were collected within the community village (7.2%) and the houses (4.8%). Some local people were cultivating some of these medicinal plants near homes for their convenience, but collecting medicinal plants in the wild during seasonal times or in case of immediate treatment was highly encouraged for efficacy as the locals believed that the plants should grow in their natural setting rather than cultivation. Scientific studies tend to support the idea of medicinal plant collection in the wild because plant secondary metabolites will be mostly expressed in the natural setting under environmental stress and conditions, whereby they could not be comparably expressed under monoculture conditions [84]. Higher levels of secondary metabolites were also reported in wild populations where plants grow slowly, unlike in much faster-growing monocultures [85].

### Plant parts used

**Fig. 2:**
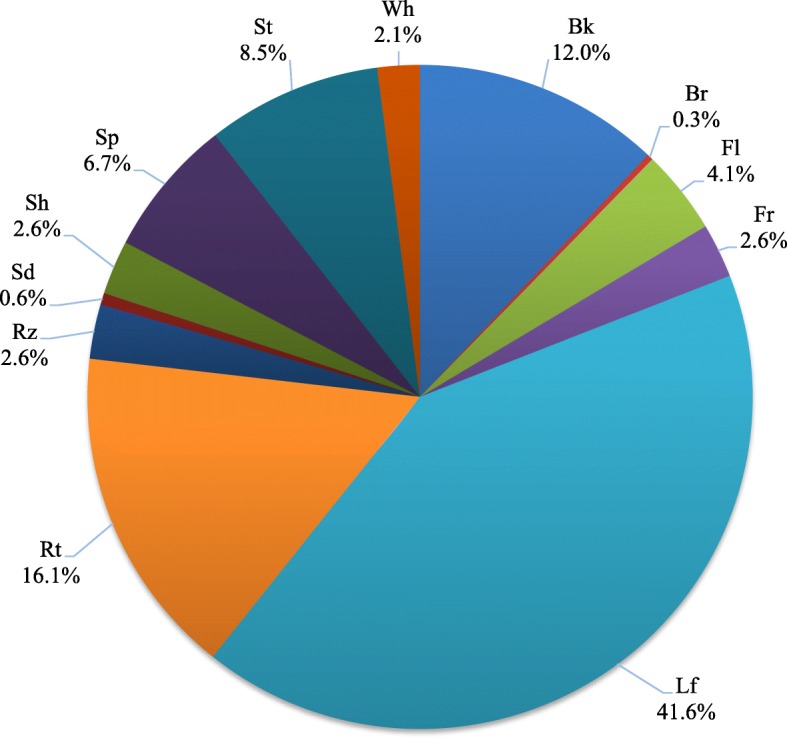
Plant parts used by the *Agusan Manobo* for medicinal application. Bk, barks; Br, branches; Fl, flowers; Fr, fruits; Lf, leaves; Rt, roots; Rz, rhizomes; Sd, seeds; Sh, shoots; Sp, sap or latex; St, stems; Wh, whole plant

All plant parts were used from different plant species against a variety of diseases. The most frequently used plant parts were the leaves (41.6%), followed by roots (16.1%), barks (12.0%), stems (8.5%), sap or latex (6.7%), and flowers (4.1%) (Fig. 2). Sometimes, more than one plant part of the same species is used in combination, like leaves, barks, stems, and roots for preparation and administration, which the locals believed to have a synergistic effect and a more effective medication.

### Preparation and administration

**Fig. 3:**
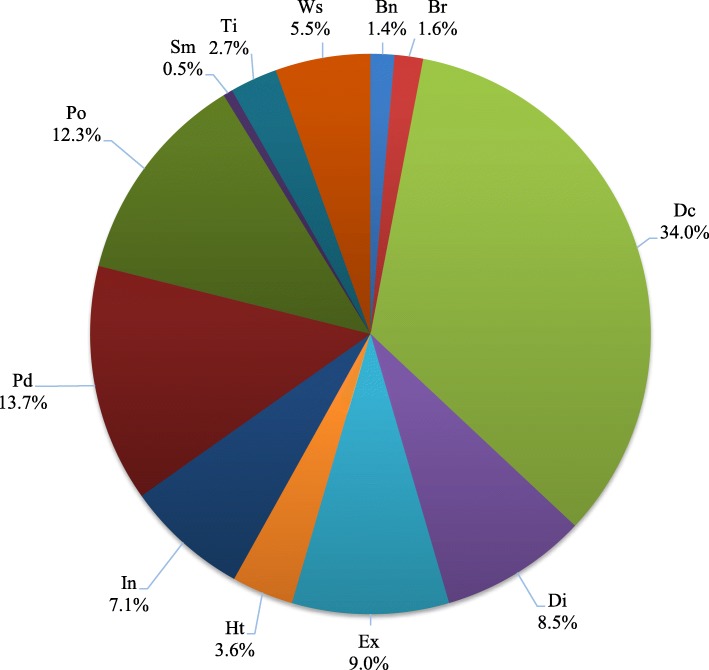
Mode of preparation of medicinal plants used by the *Agusan Manobo*. Bn, burning; Br, brewing; Dc, decoction; Di, directly applying or eating; Ex, extracting; Ht, heating or warming; In, infusion; Pd, pounding, crushing, rubbing, grinding, powdering; Po, poultice; Sm, steaming; Ti, tincture; Ws, as wash, bath, hot compress

The primary preparation method was decoction (34.0%), followed by pounding, crushing, rubbing, grinding, and powdering (13.7%); poultice (12.3%); extracting (9.0%); directly applying or eating (8.5%); infusion (7.1%); applying as wash, bath, hot compress (5.5%); heating or warming (3.6%); tincture (2.7%); brewing (1.6%); burning (1.4%); and steaming (0.5%) as depicted in Fig. 3. The more common route of administration was internal (60%) rather than external (40%). This result is contrary to the previous reports in the other Philippine major island ethnic tribes like the *Ati Negrito* community of Visayas [21] and the *Ivatan* community in Luzon [24] where the external application was more common. While external administration could be safer, according to the *Agusan Manobo*, the internal application was more common since most of their health conditions were associated internally, making decoction as their most common preparation. In cases of external diseases and illnesses, more prolonged coconut oil infusions of medicinal plant stems and barks were often applied.

### Use categories (UC)

Reported medicinal uses of plants in this study were grouped into 16 category names based on the citations of informants and the likeness to the use category (Table 3). Reported uses and diseases in medical terms were verified by the assigned local physicians and allied workers, nearby hospitals and health centers to confirm disease occurrence and epidemiology in the area. A total of 120 reported uses or diseases treated by 122 plant species were documented in the study sites.

### Use-report (UR) and use value (UV)

Both UR and UV represent the relative importance of medicinal plants for certain categorized uses or diseases. High values were considered the most important species among the *Agusan Manobo*. Five medicinal plants with the highest URs (more than 900) as well as UVs (more than 2.5) were *Anodendron borneense* (UR = 1134; UV = 3.39) in 12 categories, *Piper decumanum* (UR = 1018; UV = 3.04) in 9 categories, *Micromelum minutum* (UR = 955; UV = 2.85) in 9 categories, *Arcangelisia flava* (L.) Merr. (UR = 922; UV = 2.75) in 10 categories, and *Cinnamomum mercadoi* (UR = 908; UV = 2.71) in 8 categories, as shown in Table 4. These high UR and UV plants were the most frequently used plant species based on high fidelity level for pregnancy (FL = 88%), skin rashes and itchiness (FL = 95%), hemorrhage (FL = 97%), tumor (FL = 87%), and stomach trouble (FL = 100%), respectively, (Table 11).

The respondents consistently reported these in all study sites, but only harvested in the wild. Some other plants can be cultivated with high UVs, as shown in the top 20 species ranked by UV (Table 7). While high UV species can often be harvested for medicinal use and purpose, these important species call for conservation priority [86]. The four medicinal plants included among the top 10 recommended medicinal plants by the Department of Health (DOH) of the Philippines, were cultivated by the *Agusan Manobo* respondents within their community. These scientifically validated medicinal plants were also reported with high URs, namely “Bayabas” *Psidium guajava* L. (275) “Lagundi” *Vitex negundo* L. (475), “Gabon” *Blumea balsamifera* (L.) DC. (412), and “Tsaang gubat” *Ehretia microphylla* Lam. (336).

**Table 7:** The top 20 species ranked by use value (UV). Species which are on the top 20 lists ranked by cultural importance value (CIV) and use diversity (UD) are indicated by bold typeface in that column

Scientific name	UV	CIV	UD
*Anodendron borneense* (King & Gamble) D.J.Middleton	3.39	**3.68**	**2.22**
*Piper decumanum* L.	3.04	**3.45**	**2.06**
*Micromelum minutum* (G.Forst.) Wight & Arn.	2.85	**3.28**	**2.03**
*Arcangelisia flava* (L.) Merr.	2.75	**3.23**	**2.14**
*Cinnamomum mercadoi* S.Vidal	2.71	**3.22**	**1.93**
*Piper nigrum* L.	2.46	2.41	1.20
*Jatropha gossypifolia* L.	2.41	**2.83**	**1.94**
*Tinospora crispa* (L.) Hook. f. & Thomson	2.33	2.68	**1.95**
*Sida rhombifolia* L.	2.29	2.55	1.87
*Hellenia speciosa* (J.Koenig) Govaerts	2.22	2.58	**2.03**
*Premna odorata* Blanco	1.99	**2.94**	1.79
*Carica papaya* L.	1.97	**2.92**	1.64
*Ficus concinna* (Miq.) Miq.	1.81	2.66	1.37
*Rhinacanthus nasutus* (L.) Kurz	1.74	**2.90**	1.74
*Ficus septica* Burm.f.	1.72	2.13	1.77
*Stenomeris borneensis* Oliv.	1.61	2.36	1.70
*Pipturus arborescens* (Link) C.B.Rob.	1.61	2.25	1.58
*Ormosia macrodisca* Baker	1.56	2.36	1.56
*Orthosiphon aristatus* (Blume) Miq.	1.53	**2.96**	1.58
*Pseudelephantopus spicatus* (Juss.) Rohr	1.49	2.50	1.44

### Cultural importance value (CIV)

CIV often identifies species with diverse use-reports in different use categories, which is relatively dependent on the sum of the proportion of informants who cited the medicinal plant use. The usefulness of species based on the number of informants for each species is not only accounted for this additive index but also its versatility [47]. The top 20 species ranked by CIV included some species with high UV and UD (Table 8).

**Table 8:** The top 20 species ranked by cultural importance value (CIV). Species which are on the top 20 lists ranked by use value (UV) and use diversity (UD) are indicated by bold typeface in that column.

Scientific name	CIV	UV	UD
*Anodendron borneense* (King & Gamble) D.J.Middleton	3.68	**3.39**	**2.22**
*Piper decumanum* L.	3.45	**3.04**	**2.06**
*Micromelum minutum* (G.Forst.) Wight & Arn.	3.28	**2.85**	**2.03**
*Arcangelisia flava* (L.) Merr.	3.23	**2.75**	**2.14**
*Cinnamomum mercadoi* S.Vidal	3.22	**2.71**	**1.93**
*Andrographis paniculata* Nees	3.07	1.43	**2.09**
*Eleusine indica* (L.) Gaertn.	3.04	1.44	**2.34**
*Ficus cassidyana* Elmer	3.00	1.47	1.89
*Orthosiphon aristatus* (Blume) Miq.	2.96	**1.53**	1.58
*Premna odorata* Blanco	2.94	**1.99**	1.79
*Carica papaya* L.	2.92	**1.97**	1.64
*Rhinacanthus nasutus* (L.) Kurz	2.90	**1.74**	1.74
*Kalanchoe pinnata* (Lam.) Pers.	2.88	1.45	**2.21**
*Mangifera indica* L.	2.85	0.66	1.47
*Litsea cordata* (Jack) Hook.f.	2.83	0.92	1.79
*Jatropha gossypifolia* L.	2.83	**2.41**	**1.94**
*Mentha canadensis* L.	2.81	1.29	**2.04**
*Euphorbia hirta* L.	2.80	0.91	1.85
*Cyanthillium cinereum* (L.) H.Rob.	2.78	1.42	1.42
*Mikania cordata* (Burm.f.) B.L.Rob.	2.75	1.19	1.67

### Use diversity (UD)

UD determines medicinal plants dependent on the variety of uses in different use categories. This index considers the widespread contribution of each use category according to the number of reported diseases treated. The top 20 species with high UD did not include all high values of UV and CIV (Table 9).

**Table 9:** The top 20 species ranked by use diversity (UD). Species which are on the top 20 lists ranked by use value (UV) and cultural importance value (CIV) are indicated by bold typeface in that column

Scientific name	UD	UV	CIV
*Eleusine indica* (L.) Gaertn.	2.34	1.44	**3.04**
*Anodendron borneense* (King & Gamble) D.J.Middleton	2.22	**3.39**	**3.68**
*Kalanchoe pinnata* (Lam.) Pers.	2.21	1.45	**2.88**
*Swietenia mahagoni* (L.) Jacq.	2.14	1.00	2.29
*Arcangelisia flava* (L.) Merr.	2.14	**2.75**	**3.23**
*Andrographis paniculata* Nees	2.09	1.43	**3.07**
*Ocimum basilicum* L.	2.09	1.15	2.33
*Piper decumanum* L.	2.06	**3.04**	**3.45**
*Amaranthus spinosus* L.	2.06	0.63	2.75
*Mentha canadensis* L.	2.04	1.29	**2.81**
*Alstonia macrophylla* Wall. ex G.Don	2.04	1.15	2.71
*Micromelum minutum* (G.Forst.) Wight & Arn.	2.03	**2.85**	**3.28**
*Hellenia speciosa* (J.Koenig) Govaerts	2.03	**2.22**	2.58
*Annona muricata* L.	2.02	0.62	2.17
*Phyllanthus amarus* Schumach. & Thonn.	2.01	1.37	2.35
*Abroma augusta* (L.) L.f.	1.98	0.98	2.69
*Mimosa pudica* L.	1.97	1.06	2.29
*Tinospora crispa* (L.) Hook. f. & Thomson	1.95	**2.33**	2.68
*Jatropha gossypifolia* L.	1.94	**2.41**	**2.83**
*Cinnamomum mercadoi* S.Vidal	1.93	**2.71**	**3.22**

### Correlation of the basic values and indices

Table 10 presents the Spearman correlations among all the five variables used to quantify ethnopharmacological data. All correlations were moderate to strongly positive and significant at *p* < 0.01 (*n* = 125). That is, as one variable increases, the other also increases. Of all the variables, UV is entirely dependent on UR (1.00), while UD is highly dependent on UC (0.97). However, the subjectivity of selection criteria among the use categories was avoided as the researcher consulted with physicians and other medical experts in the locality. The correlation index between UV and CIV was quite high (0.73), meaning that the relative importance of medicinal plants used among the *Agusan Manobo* was relatively dependent on the number of use mentions among the key informants as counted in UR. An interesting point that appeared to corroborate these data is that the number of UR was positively correlated (0.71–1.00), among other basic values and indices. These variables were correlated with the number of uses for a particular ailment and the number of categories considered. Thus, it can be argued that the relative importance of medicinal plants documented in this study was relatively dependent at least, on the number of use-reports among the key informants and the number of use categories following an objective manner. Despite the advantages and uses of these values and indices in determining the relative importance and usefulness of medicinal plants, it is practical to note that no single index can give information about the complete picture of plant importance.

**Table 10:** Spearman rank order correlations among all five variables: basic values and indices

	UC	UV	CIV	UD
UR	0.74	1.00	0.73	0.71
UC		0.74	0.71	0.97
UV			0.73	0.71
CIV				0.69

All the correlations are significant at *p* < 0.01 (*n* = 125)

### Informant consensus factor (ICF)

ICF measures the agreement among informants on the use of plant species for a particular purpose or disease category. While the agreement among the key informants varies in different categories, the ICF values are all greater than or equal to 0.97 (Table 3). These results showed that the exchange of information could be evident among the *Agusan Manobo* community on their medicinal plant uses and practices. Among the 16 use categories, four categories, namely diseases of the digestive system (DDS), diseases of the skin (DOS), abnormal signs and symptoms (ASS), and other problems of external causes (OEC) had the highest ICF value of 0.99.

### Fidelity level (FL)

FL implies the most preferred medicinal plant for a particular disease or purpose. FL value ranges from 1 to 100% depending on the URs cited by the informants for a given species for a particular ailment. Seven species were found with the maximum FL of 100%, including the identified species with the highest number of use mentions, *Carica papaya*, *Premna odorata*, *Cinnamomum mercadoi*, *Tinospora crispa*, *Ficus concinna*, *Piper decumanum*, and *Pipturus arborescens* which are used for dengue fever, cough with phlegm, stomach trouble, joint pain, fracture and dislocation, anesthetic, and herpes simplex, respectively (Table 11).

**Table 11:** The relative healing potential of the top 20 most cited medicinal plants used against particular disease

No.	Scientific name	Particular use or disease	Ip	Iu	FL%
1	*Carica papaya* L.	Dengue fever	158	158	100
2	*Premna odorata* Blanco	Cough with phlegm	238	238	100
3	*Cinnamomum mercadoi* S.Vidal	Stomach trouble	223	223	100
4	*Tinospora crispa* (L.) Hook. f. & Thomson	Joint pain	157	157	100
5	*Ficus concinna* (Miq.) Miq.	Fracture and dislocation	41	41	100
6	*Piper decumanum* L.	Anesthetic	68	68	100
7	*Pipturus arborescens (*Link) C.B.Rob.	Herpes simplex	59	59	100
8	*Rhinacanthus nasutus* (L.) Kurz	Nervous breakdown	44	45	98
9	*Stenomeris borneensis* Oliv.	Urinary bladder swelling	133	136	98
10	*Micromelum minutum* (G.Forst.) Wight & Arn.	Hemorrhage	70	72	97
11	*Piper nigrum* L.	Skin rashes and itchiness	203	214	95
12	*Jatropha gossypifolia* L.	Discharging ear	59	63	94
13	*Orthosiphon aristatus* (Blume) Miq.	Diabetes	68	72	94
14	*Ormosia macrodisca* Baker	Atherosclerosis	63	69	91
15	*Sida rhombifolia* L.	Cramp and spasm	71	79	90
16	*Pseudelephantopus spicatus* (Juss.) Rohr	Urinary tract infection	85	95	89
17	*Anodendron borneense* (King & Gamble) D.J.Middleton	Pregnancy	38	43	88
18	*Arcangelisia flava* (L.) Merr.	Tumor	73	84	87
19	*Hellenia speciosa* (J.Koenig) Govaerts	Goiter	44	52	85
20	*Ficus septica* Burm.f.	Eye problem	32	39	82

*FL%* percentage of fidelity level, *Ip* the number of informants who independently cited the use of a species for a particular use or disease, *Iu* the total number of informants who mentioned the plant for any use or purpose regardless of category

### Jaccard’s similarity index (JI)

**Fig. 4:**
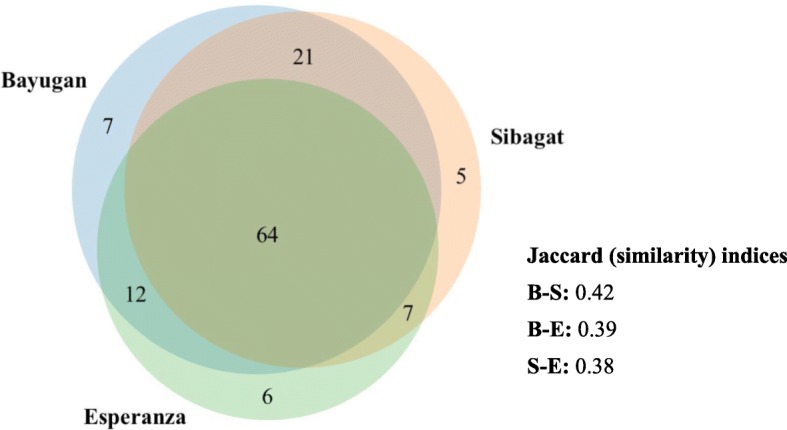
Overlap in the medicinal plants collected in the three studied localities (city of Bayugan and the municipalities of Sibagat and Esperanza)

This is the first ethnopharmacological or ethnobotanical study of indigenous peoples in the province of Agusan del Sur. The variation of the medicinal plants used among the three studied localities was shown in JI (Fig. 4). The most overlap of the obtained data and the Jaccard index (similarity) was between the city of Bayugan and the municipality of Sibagat (JI = 0.42), and the least one was between both municipalities of Esperanza and Sibagat (0.38). However, the degree of similarity among the three adjacent localities was proximate with JI ranged from 0.38 to 0.42. While JI conveyed a similarity index ca. 39.7%, the actual overlap is 52.5% (64 species cited among the localities). This similarity could be observed on their comparable ecological types being upland and well-drained areas and due to the active exchange of information on the uses of medicinal plants among the communities during monthly social meetings and preparations in the province of Agusan del Sur.

### Dosage, frequency, and experienced adverse or side effects of using medicinal plants

For a detailed ethnopharmacological study, it is essential to consider the therapeutic use, medication action, and possible side effects. This study involved documenting the quantity or dosage, administration frequency, and experienced adverse or side effects, as shown in Table 4. A particular number of plant parts were followed in their mode of preparation. Having leaves as the most frequently used medicinal plant part, 3–5 leaves (or at least an odd number) of decocted, heated, and pounded leaves should be applied. Most of the medicinal plants (82%) were reported by the key informants with no experience of adverse or side effects, while 18% of medicinal plants were experienced with adverse or side effects. There were seven medicinal plants reported to cause abortion in pregnant women once taken or applied. Other listed medicinal plants, when taken in excess, can cause other adverse or side effects. Four of these medicinal plants can cause anemia, dizziness, and weakening, while other plants can cause acid reflux and hypocupremia, burn, and allergy and are even poisonous when eaten or applied. Other reported cases concern excessive intake, which can cause blood viscosity, intestinal weakening, thrombocytopenia, and abnormalities in lactating mothers. These reported adverse or side effects were verified by the attending local medical practitioners and allied medical workers during their hospital visits and in times of emergency. It can be argued that not all medicinal plants used by the tribe are safe for use with no side effects. Thus, it is essential to obtain the reported adverse effects or possible side effects of cited medicinal plants by the informants in all ethnopharmacological studies like this.

## Discussion

This ethnopharmacological documentation recorded a total of 122 medicinal plant species belonging to 108 genera and 51 families across 16 use or disease categories. The majority of medicinal plants are trees (36%) and herbs (33%), which are mostly found in the wild, while some are cultivated. These are followed by 17% shrubs, 11% climbers, 2% grasses, and 1% ferns. The highest percentage of medicinal trees documented in this study is parallel with the earlier ethnobotanical studies [21, 87]. The highest frequency of using leaves and aerial plant organs was also reported in several ethnobotanical studies in the Philippines [21, 24, 25, 87–90] and other countries [91–93]. The highest frequency of decoction for preparation and administration is similar to previous ethnobotanical investigations [21, 87–90].

Lamiaceae was the most represented family with 12 species, followed by Asteraceae with 11, Moraceae with eight species, and Fabaceae with six species. This result is contrary to previous ethnobotanical studies in which Asteraceae were the most represented family [24, 88–90]. The Lamiaceae (mint family) possess a wide variety of ornamental, medicinal, and aromatic plants producing essential oils that are used in traditional and modern medicine, food, cosmetics, and pharmaceutical industry [94]. This family is known for effective pain modulation with potential analgesic or antinociceptive effects, which includes several aromatic medicinal spices like mint, oregano, basil, and rosemary [95]. Asteraceae (the aster, daisy, composite, or sunflower family) are the largest family of flowering plants which were reported to have pharmacological activities such as antitumor, antibacterial, antifungal, and anti-inflammatory [96] containing phytochemical compounds such as polyphenols, flavonoids, and diterpenoids [97, 98]. The Moraceae (fig family) was reported to have wide variety of chemical constituents with potential biological activities as previously investigated by [99] in *Ficus racemosa* L., and [100] in *Ficus carica* L., and [101] in *Ficus benjamina* L. Fabaceae (pea family) which is the third largest family also contain various bioactive constituents with potential pharmacological and toxicological effects [102]. A member of this family which has long been cultivated and introduced in the Philippines, *Gliricidia sepium* (Jacq.) Kunth ex Steud., was investigated to have antimicrobial and antioxidant activities, as well as several phytochemicals present [13].

The Department of Health (DOH) of the Philippines has continually endorsed 10 medicinal plant species in its traditional health maintenance program: (1) *Cassia alata* L., (2) *Momordica charantia* L., (3) *Allium sativum* L., (4) *Psidium guajava* L., (5) *Vitex negundo* L., (6) *Quisqualis indica* L., (7) *Blumea balsamifera* (L.) DC., (8) *Ehretia microphylla* Lam., (9) *Peperomia pellucida* (L.) Kunth, and (10) *Clinopodium douglasii* (Benth.) Kuntze*.* Of all these 10 recommended and clinically tested medicinal plants, four species were included in this survey.

Apparently, the societal gaps which differentiate educational level, gender, position, occupation, and age among the *Manobo* indigenous community may result in the disappearance of their medicinal plant knowledge and traditional practices. While there was no significant difference in their medicinal plant knowledge in different locations, it is still highly important to document their medicinal plant knowledge to perpetuate their cultural tradition and medicinal practices, as well as protect and conserve these important plant genetic resources.

Many ethnobotanical studies include vernacular names as part of the putative identification. While vernacular names are useful in ethnopharmacology, pharmacognosy, and pharmacovigilance [83, 103], reliance on these vernacular names for species identification and classification can cause ambiguity and incorrect identification resulting to research invalidation [104]. DNA-based identification is a useful tool for accurate species identification. Correct identification of a medicinal plant should be examined using molecular data [105] for consistency of species and pharmacological investigations of natural products [106]. Although plant-based drug discovery from ethnobotanical data provides future drug leads, authentication of the plant material is a great challenge and opportunity [107].

### Comparison with previous ethnobotanical studies

Several ethnobotanical and ethnomedicinal studies were conducted in the Philippines, but few involve quantitative analyses in their studies. The majority of ethnobotanical studies conducted in the Philippines purposively selected key informants who are just knowledgeable of their medicinal plants like residents, traditional healers, herbalists, gardeners, traders, and elders, but a limited count of researches focused on specific IPs or tribal communities in the country.

Among the three major islands in the Philippines (Luzon, Visayas, and Mindanao), the island of Mindanao is still underdocumented despite its largest population of indigenous cultural communities/indigenous peoples (ICCs/IPs) in the country. In Luzon, four indigenous groups were documented, namely the *Kalanguya* tribe in Tinoc, Ifugao [108]; the *Ivatan* in Batan Island Batanes [24]; the *Ayta* in Dinalupihan, Bataan [109]; and the *Ilongot-Eǵongot* in Maria Aurora, Aurora [110], communities. The plant utilization among local communities was also documented by [25] in Kabayan, Benguet Province, namely *Ibaloi*, *Kankanaey* and *Kalanguya* in addition to the earlier recorded tribes such as the *Negritos* [111], the *Tasadays* [112, 113], the *Ifugao* [114, 115] and the *Bontoc* [116]. Other studies of cultural communities involve indigenous knowledge and practices for sustainable management like the *Ifugao* forests in Cordillera, Philippines [117].

In Visayas, only the *Ati Negrito* of Guimaras island [21], while in Mindanao, three tribes were studied, namely the *Higaonon* tribe of Iligan City [88], *Subanen* tribe of Dumingag, Zamboanga del Sur [89]; *Muslim Maranaos* of Iligan City [90]; *Subanen* tribe of Lapuyan, Zamboanga del Sur [87]; and *Tagabawa* tribe of Davao del Sur [118]. Of all reported ethnobotanical studies in Mindanao, this is the first study utilizing detailed quantitative analysis of relative importance, effectivity consensus, correlation of indices, and the extent of the potential use of each medicinal plant species among the ICCs/IPs. Moreover, this study also integrated molecular confirmation for the first time applying multiple universal markers and coalescing a priori and a posteriori data for accurate species identification to resolve complex plant local or vernacular names and sterile or non-reproductive plant specimens.

In comparison with existing ethnobotanical studies in the Philippines, a novel plant medicinal use was recorded, namely *Anodendron borneense* with no existing records of ethnobotanical and pharmacological investigations in the world to date. The ethnopharmacological profile of this medicinal plant is a novel finding in this study, which is consistently on the top list among the values or indices used (UR, UV, and CIV), which is only known among the *Agusan Manobo* in the province of Agusan del Sur, Philippines. Incorporating data of experienced adverse or side effects in this study introduces a more detailed ethnopharmacological documentation in the Philippines, which could be a reference material for future ethnomedicinal, biological, and pharmacological studies.

### Limitations of the present study

Ethnobotanical research broadly encompasses like ethnopharmacology, which involves field-based investigations. However, most of the remote areas and barangays in various municipalities and cities of the Philippines were not always safe from rebels and communists against the Philippine government. Majority of the *Manobo* tribes documented here live in far-flung hinterlands, remote upland areas alongside rivers, valleys, and creeks having security threats from the rebel movement known as the New People's Army (NPA). Study sites included here obtained security clearance from the provincial and local government administrations to ensure safety and accessibility in the area, and the availability of key informants on the actual documentation and field walks. Language barriers were barely encountered since most respondents could speak the national *Filipino* language and/or the regional *Cebuano* or *Visayan* language aside from their *Minanubu* dialect. Phenology and year-round seasonal variations are essential factors to consider for accurate observation of the plant and collection of specimens with complete reproductive parts. Some respondents are sometimes unwilling to share their medicinal plant knowledge with others due to their previous experience being taken advantage of by business-related parties of drug and pharmaceutical companies. It was also observed that most respondents are becoming educated with the help of government education programs for IPs, which made them more resistant to allowing themselves to be the subject of study by visitors and outsiders.

In spite of that, it is very important to gain trust, confidence, and respect among the *Agusan Manobo* community by embracing their rich cultural tradition through ritual observation and tribal immersion within their community. Although they maintain secrecy about their medicinal plant use and knowledge, it is also beneficial to practice keeping their knowledge from possible overexploitation of their medicinal plant resources. This study is the first in the country documenting the rich ethnopharmacological practices of indigenous tribes coupled with integrative molecular confirmation of medicinal plants used. It is highly important to recognize the role of indigenous cultural communities/indigenous peoples (ICCs/IPs) in the Philippines for shared information of ethnopharmacological practices for future preservation of knowledge and conservation priorities of their plant genetic resources. This will benefit their children and future generations before their knowledge becomes lost and forgotten.

### Research highlights

1. The current study revealed the rich ethnopharmacological practices, medicinal plant uses, and knowledge of the *Manobo* tribe in Agusan del Sur, Philippines.
2. Exchange of information among the *Agusan Manobo* communities was observed in different localities; however, the younger generation has a potential decline of interest due to their acquaintance of over-the-counter drugs and modern medicines.
3. This study reinforced the application of integrative molecular confirmation for medicinal plant species lacking reproductive parts upon collection and/or unidentifiable by present morphology (sterile or non-reproductive) plant material.
4. Novel medicinal use and some new ethnopharmacological information of medicinal plants were reported in this study.
5. The consolidated data of this quantitative ethnopharmacology study contributes to the repository of medicinal plant knowledge and the rich source of information for scientists, physicians, and experts such as botanists, taxonomists, phytochemists, pharmacists, environmentalists, conservation biologists, medical doctors, and allied professionals.

## Conclusion

This study concluded the culturally rich ethnomedicinal knowledge and ethnopharmacological practices of the *Manobo* tribe in Agusan del Sur, Philippines. The results of the study revealed a high diversity of medicinal plants used by the *Agusan Manobo* with 122 species utilized in 16 use categories. Like any other ethnolinguistic indigenous group in the country, traditional knowledge may be lost or forgotten due to possible migration, acculturation, and declining interest of the younger generation in response to the increasing availability of commercial over-the-counter medicine. Their medicinal plants are known by a limited number of individuals, mostly by their healers, elders, and tribal officials. This quantitative ethnopharmacological documentation is the first to show the high consensus and relative importance of medicinal plants used by the *Agusan Manobo* and provides molecular confirmation of their medicinal plant species with uncertain identity. The combined quantitative ethnopharmacological documentation and species confirmation using an integrative molecular approach of medicinal plants used in traditional medicine is a breakthrough for obtaining more detailed and comprehensive findings that will be a valuable contribution to the repository of knowledge. The findings of this study will serve as reference material for future systematic, biochemical, and pharmacological studies. While the findings of this study are promising, regarding new potential therapeutic agents for healthcare improvement, it is of utmost concern to reconsider important medicinal plant species for conservation priorities as part of the government programs and initiatives to perpetuate the national and world heritage of traditional knowledge on medicinal plants used by many diverse cultural communities.

## Supplementary information

**Additional file 1: **Semi-structured questionnaire with *Manobo* dialect (Minanubu) translation

**Additional file 2:** Fasta file of ITS (nrDNA) sequences

**Additional file 3:** Fasta file of matK (cpDNA) sequences

**Additional file 4:** Fasta file of psbA-trnH (cpDNA) sequences

**Additional file 5:** Fasta file of trnL-F (cpDNA) sequences

## Data Availability

The authors declare that sequencing data of 24 species identified supporting the findings of this study are available within the article and its supplementary information files.
