# Supplementary material for: Quantitative ethnopharmacological documentation and molecular confirmation of medicinal plants used by the Manobo tribe of Agusan del Sur, Philippines
Source: J Ethnobiol Ethnomed. 2020 Mar 5;16:14. doi: 10.1186/s13002-020-00363-7 (PMC7227330; doi:10.1186/s13002-020-00363-7)
Supplement: Supplementary file 1 — Additional file 1: Semi-structured questionnaire with Manobo dialect (Minanubu) translation [file 13002_2020_363_MOESM1_ESM.docx]

**Additional file 1:** Semi-structured questionnaire with *Manobo* dialect (Minanubu) translation

**Una nga Parte: Datus sa Kaugalingon**

*Part I: Personal Profile*

**Palihog ug sulat sa inyong tubag sa kada pangutana.**

*Please answer all that apply to you*

**Pangalan** *Name*: **________________________________________________________**

**Edad** *Age:* **____________**

**Tawhanun** *Sex:* **________Lalaki** Male: **____________Babae** *Female***: ____________**

**Edukasyon** *Education:* **_________________________**

**Estado Sibil** *Civil Status:* **Minyo** *Married* **_________** **Dili Minyo** *Single*: **__________**

**Puy-anan** *Address:* **_____________________________________________________**

**Trabaho/Panginabuhi** *Occupation:* **________________________________________**

**Ikaduhang Parte: Problema sa Panglawas ug Tambal nga Tanum**

*Part 2: Health Problem and Medicinal Plants Used*

1. **Nocoy tue (unsa) mgo (ang) casagare nue mgo sakit duon tue panimayoy?**

*What is/are the common health problem/s that you experienced in your household?*

1. **Unsa imong gibuhat o aha ka miadto sa miaging nagkasakit ka o imong kauban sa pamilya?** *The last time you, or somebody in your family had health problem, what did you do?*
   1. **Naadtog dokto**r *Consulted local physician or medical doctor*
   2. **Nangayog tabang sa mananambal o sa uban** *Sought healer’s or mystical person’s help*
   3. **Nag gamit ug tambal nga tanom** *Used medicinal plant/s*
2. **Unsa nga mga tanom inyong gigamit pagpanambal?**

*What is (are) the plant(s) you use?*

1. **Parte sa tanom nga gigamit panambal.** *Part(s) of plant(s) used to prepare the remedy*
   1. **Panit** *bark*
   2. **Kahoy** *stem*
   3. **Bagon** *vine*
   4. **Dahon** *leaf*
   5. **Ugat** *root*
   6. **Sanga** *branch*
   7. **Liso** *seed*
   8. **Prutas** *fruit*
   9. **Bulak** *flower*
   10. **Duga** *sap or latex*
   11. **Uban pa, pakisulat** *Others, please write ­*_____________________________________
2. **Porma sa Pagpanambal** *Administration Forms*
   1. **Gihumol sa lana** *Infusion*
   2. **Gihumol sa bino** *Tincture*
   3. **Gilaga** *Decoction*
   4. **Gidukdok** *Pounded*
   5. **Gidugmok** *Crushed*
   6. **Gihumolan sa tubig** *Aqueous/Diluted*
   7. **Gipainitan sa kalayo** *Heated/Warmed*
   8. **Gipabugnawan** *Cooled*
   9. **Hilaw** *Raw*
   10. **Gipaduga** *Juice*
   11. **Uban pa, pakisulat** *Others, please write ­***_________________________________**
3. **Pamaagi sa Pagpanambal** *Administration Mode*

**Tambal Sulod sa Lawas** *Internal Application*

- 1. **Ginainom** *Oral Absorption*
  2. **Ginasimhut/Paalisngaw** *Inhalation*
  3. **Gipatakan** *Instillation*
  4. **Gihumulan** *Apply Dipped Cotton*
  5. **Uban pa, pakisulat** *Others, please write ­***____________________________________**

**Tambal Gawas sa Lawas** *External Application*

- 1. **Pahid** *Topical Application*
  2. **Gihumulan** *Apply Dipped Cotton*
  3. **Haplas/Hilot** *Rub/Massage*
  4. **Ginaligo** *Bath*
  5. **Gipatuluan** *Drops*
  6. **Ginataplak/Ginabutang** *Poultice*
  7. **Uban pa, pakisulat** *Others, please write ­***____________________________________**

1. **Unsa kadaghan ang ibutang: (sakto nga kadaghanon)**

*Quantity or dosage of remedy taken each time: (precise as to the quantity)*

1. **Kapila sa isa ka adlaw butangan? (insakto nga oras butangan).**

*How many times a day? (precise number of times per day of application)*

1. **Pila ka adlaw butangan?**

*How many days of application?*

1. **Naa ba ni siyay dili maayo nga epekto? Unsa man ang mga makadaot na epekto?**

*Are there some adverse or side effects? What are some adverse or side effects?*

- 1. **Wala** *None* ________
  2. **Oo** *Yes*. __________

**Kung oo, unsa?** *If yes, what is/are the adverse or side?*

*__________________________________________*

*__________________________________________*

*__________________________________________*

- 1. **Wala kabalo** *Does not know* _________

1. **Andieka camaan tue pag gamit tue binisaya nue tanum?**

*With whom did you learn the use of medicinal plants?*

- 1. **Mgo Ginikanan** *Parents*
  2. **Mgo Kasuonan** *Relatives*
  3. **Mgo Kadumahan Nue Mgo Tribo** *Fellow tribe band*
  4. **Sa Komunidad** *Community*
  5. **Kaugalingon Nue Pagkaaman** *Self-discovery*
  6. **Uban pa, Pakisulat** *Others, please write***____________________________________**

1. **Andieka nue pumuduta sikan mga tambay nue tanum?** *Where did you get the plant?*
   1. **Duon Tue Bayoy** *Within the house*
   2. **Duon Tue Mgo Kasigpit** *In the community*
   3. **Duon Tue Mgo Goyanga** *In the woods or forest*
   4. **Kinahan Ug Biyahe Ka Tue Tunga Tue Adoy Oh Sungo Adoy** *Need to travel for ½ or 1 day*
   5. **Bulihon Tue Yain Lugar** *Buy on other places*
   6. **Aha pa? Pakiapil** *Where else? Please mention:* **______________________________**
2. **Tigpanguha nga panahon sa tamabal nga tanom** *Gathering season and/or hour*
   1. **Kada adlaw** daily
   2. **Kada semana** weekly
   3. **Kada bulan** monthly
   4. **Kada tuig** yearly
   5. **Uban pa, pakisulat** *Others, please write* **____________________________________**
